# Diverse Microorganisms in Sediment and Groundwater Are Implicated in Extracellular Redox Processes Based on Genomic Analysis of Bioanode Communities

**DOI:** 10.3389/fmicb.2020.01694

**Published:** 2020-07-28

**Authors:** Tyler J. Arbour, Benjamin Gilbert, Jillian F. Banfield

**Affiliations:** ^1^Department of Earth and Planetary Science, University of California, Berkeley, Berkeley, CA, United States; ^2^Energy Geosciences Division, Lawrence Berkeley National Laboratory, Berkeley, CA, United States; ^3^Department of Environmental Science, Policy, and Management, University of California, Berkeley, Berkeley, CA, United States

**Keywords:** extracellular electron transfer, multiheme cytochromes, bioelectrochemical systems, microbial electrochemical cells, *Geobacter*, metagenomics, porin-cytochrome complex, e-pili

## Abstract

Extracellular electron transfer (EET) between microbes and iron minerals, and syntrophically between species, is a widespread process affecting biogeochemical cycles and microbial ecology. The distribution of this capacity among microbial taxa, and the thermodynamic controls on EET in complex microbial communities, are not fully known. Microbial electrochemical cells (MXCs), in which electrodes serve as the electron acceptor or donor, provide a powerful approach to enrich for organisms capable of EET and to study their metabolism. We used MXCs coupled with genome-resolved metagenomics to investigate the capacity for EET in microorganisms present in a well-studied aquifer near Rifle, CO. Electroactive biofilms were established and maintained for almost 4 years on anodes poised mostly at −0.2 to −0.25 V vs. SHE, a range that mimics the redox potential of iron-oxide minerals, using acetate as the sole carbon source. Here we report the metagenomic characterization of anode-biofilm and planktonic microbial communities from samples collected at timepoints across the study period. From two biofilm and 26 planktonic samples we reconstructed draft-quality and near-complete genomes for 84 bacteria and 2 archaea that represent the majority of organisms present. A novel *Geobacter* sp. with at least 72 putative multiheme *c*-type cytochromes (MHCs) was the dominant electrode-attached organism. However, a diverse range of other electrode-associated organisms also harbored putative MHCs with at least 10 heme-binding motifs, as well as porin-cytochrome complexes and e-pili, including Actinobacteria, Ignavibacteria, Chloroflexi, Acidobacteria, Firmicutes, Beta- and Gammaproteobacteria. Our results identify a small subset of the thousands of organisms previously detected in the Rifle aquifer that may have the potential to mediate mineral redox transformations.

## Introduction

Some members of subsurface microbial communities rely on extracellular minerals as electron acceptors for their metabolism. In other cases, organisms themselves serve as the electron donor or acceptor in metabolic electrochemical syntrophy (Stams et al., 2006; Kato et al., 2012; Shrestha et al., 2013; McGlynn et al., 2015). These biochemical redox interactions are known as extracellular electron transfer (EET) (Shi et al., 2016; Tremblay et al., 2017), and help drive geochemical changes and cycles that shape Earth’s bio- and geospheres (Weber et al., 2006). In most known cases of microbial EET, specialized electron-transfer proteins known as multiheme *c*-type cytochromes (MHCs) play a vital role (Shi et al., 2016). In Gram-negative bacteria, genes encoding MHCs are often found adjacent to, and therefore co-expressed with, genes for outer-membrane porin proteins, which together form porin-cytochrome complexes (PCCs) capable of transmitting electrons across the cell outer membrane (Hartshorne et al., 2009; Shi et al., 2014). Beyond the cell membrane, other biological components shown to form micrometer-long bacterial “nanowires” include type-IV “e-pili” with a high density of aromatic amino acid residues that may allow for delocalized electron flow (Lovley and Walker, 2019), and polymerized chains composed entirely of the hexaheme cytochrome OmcS (Wang et al., 2019).

Most of the knowledge gained about EET comes from studies of the first metal-respiring bacteria to be isolated, *Geobacter* (species *metallireducens* and *sulfurreducens*) of the Deltaproteobacteria and *Shewanella oneidensis* of the Gammaproteobacteria (Lovley et al., 1988; Myers and Nealson, 1988; Caccavo et al., 1994), which became and remain the model organisms for EET. Complete genome sequences and genetic systems for these organisms have been publicly available since the early 2000’s, allowing detailed physiological investigations (Heidelberg et al., 2002; Methé et al., 2003).

Microbes able to perform EET have now been isolated from many additional phylogenetic groups [reviewed in Gregory and Holmes (2011), Koch and Harnisch (2016)], and EET is increasingly recognized as a widespread phenomenon with deep evolutionary history. In their review, Koch and Harnisch (2016) highlighted 94 species (representing eight phyla and fifteen classes) for which convincing evidence of electroactivity has been reported. However, lacking complete genomes, few genetic details are known about the majority of these organisms, and therefore about the mechanisms underlying their EET capability. Furthermore, experiments in which electrodes or redox-active minerals were used as electron acceptors (or donors) to target electroactive microbes have consistently resulted in diverse, stable mixed-species consortia (Lee et al., 2003; Holmes et al., 2004; Jung and Regan, 2007; White et al., 2009; Yates et al., 2012). Yet, few of the species commonly present have been isolated in pure culture. This is almost certainly due to the fact that in nature microorganisms exist and function as members of a community, relying on a number of complex interspecies interactions (Little et al., 2008; Abreu and Taga, 2016; Anantharaman et al., 2016).

Microbial electrochemical cells (MXCs) in combination with genome-resolved metagenomics provide a powerful approach to overcome the above challenges. The MXC, in which an electrode plays the role of the mineral (or microbial) electron acceptor, is a proven system for cultivating electroactive microbes, and metagenomics can yield complete genomic information to identify members of the community and predict metabolic functions. While electrodes may only approximate natural mineral electron acceptors (Levar et al., 2017), they reduce or eliminate the intrinsic heterogeneity in redox potential and other properties that influence their bioavailability (Thamdrup, 2000; Navrotsky et al., 2008; Bonneville et al., 2009). In an MXC, the voltage at the electrode can be precisely controlled, allowing for exploration of specific redox niches (Bond, 2010; Jung and Regan, 2011). These bioelectrochemical systems therefore provide a way to enrich specifically for electroactive microbes, and typically result in high cell densities in both the planktonic medium and electrode-attached biofilms (e.g., Holmes et al., 2004).

In this study, we coupled enrichment using MXCs with genome-resolved metagenomics. The source of the inoculum was a major subsurface field research site located in Rifle, CO, United States. Sediment and groundwater from this site have been the subject of many prior metagenomic studies (Wrighton et al., 2012, 2014; Castelle et al., 2013; Hug et al., 2013, 2016; Brown et al., 2015; Anantharaman et al., 2016; Jewell et al., 2016). This allowed us to compare our results to genomic information from the Rifle aquifer, making it possible to place many of the organisms enriched in the MXCs into their environmental context. The identification and detailed characterization of 31 porin-cytochrome complexes from 12 genomes representing four taxonomic Classes, as well as putative e-pili from a similarly broad array of taxa, will be of particular interest to other researchers in the field. Overall, the results implicate a diverse consortium of bacteria (and a few archaea) in electrode-based and, by proxy, mineral-based respiration.

## Materials and Methods

### Inoculation and Operation of Microbial Electrochemical Cells

Custom electrochemical cells with duplicate anodic chambers on either side of a central, shared cathodic chamber were constructed for this study (Supplementary Figure S1). Complete details of the cell setup and components are given in the Supplementary Methods. Briefly, unpolished graphite-block electrodes (2.5 cm × 2.5 cm × 1.0 cm, McMaster-Carr, Chicago, IL, United States) served as anodes, and platinum-mesh (∼1.5 cm × 1.0 cm) as cathode. Phosphate-buffered medium was used in both anodic and cathodic chambers, and contained the following per 1 L: NaH2PO4 plus Na2HPO4 (anhydrous; 0.38 g and 0.97 g, resp. for 10.0 mM total phosphate buffer), KCl (0.10 g, 1.3 mM), NH4Cl (0.25 g, 4.7 mM). Trace minerals and vitamins were added from 100× stock solutions (10 mL per L) and contained the following per L distilled water. Minerals (pH to 6.5 with KOH): 1.5 g nitrilotriacetic acid disodium salt, 3 g MgSO47H2O, 0.5 g MnSO42H2O, 1.0 g NaCl, 0.1 g FeSO47H2O, 0.1 g CoCl22H2O, 0.1 g CaCl22H2O, 0.1 g ZnSO4, 0.025 g Na2MoO4, 0.024 g NiCl26H2O, 0.01 g CuSO45H2O, 0.01 g AlK(SO4)2, 0.01 g H3BO3. Vitamins: 2 mg biotin, 2 mg folic acid, 10 mg pyridoxine-HCl, 5 mg thiamine-HCl, 5 mg riboflavin, 5 mg nicotinic acid, 5 mg DL-calcium pantothenate, 0.1 mg vitamin B12, 5 mg p-aminobenzoic acid, 5 mg lipoic acid (Balch et al., 1979; Lovley et al., 1984). Nafion 117 (DuPont, Wilmington, DE, United States) was used as the proton exchange membrane separating anodic and cathodic chambers. Anaerobic conditions were maintained in anodic chambers by constant flushing with ultrahigh-purity N_2_ gas passed through an oxygen trap (Grace Alltech OxiClear, Thermo Fisher Scientific, Waltham, MA, United States) and 0.2-μm filters. Oxygen served as the electron acceptor at the cathode and was supplied by a steady stream of humidified air passed through a 0.2-μm filter.

Anodic chambers of a first microbial electrochemical cell, MXC-1, were inoculated with a sediment-groundwater slurry obtained from the United States Department of Energy’s Subsurface Systems Scientific Focus Area 2.0 site in Rifle, CO, United States (henceforth referred to as the Rifle site). This sediment sample (BH 3.2.11) was recovered from alluvium at the bottom of a freshly dug trench with a depth of ca. 3.5 m below ground surface (co-located with the depth to groundwater) in March 2011. The trench was located in an area not subjected to prior acetate amendments. Alluvium was passed through a 2.5 mm sieve, added to no-headspace Mason jars and saturated with groundwater from the trench. Samples were stored at 4°C in the dark for 6.5 months. For inoculation, the sediment-groundwater slurry was shaken to mix and then quickly introduced into anodic chambers through the MXC sampling ports using a sterile spatula for the coarser sediment grains, and a sterile, N_2_-flushed syringe for the fine-grain groundwater suspension. Acetate was used as electron donor because it is a well-known fermentation product linked to microbial community function at Rifle and in general (Wrighton et al., 2014), as well as a preferred substrate of electroactive *Geobacter* species. Further, acetate has been used during *in situ* field experiments at the Rifle site (Williams et al., 2011; Handley et al., 2013). A sterile, anaerobic stock solution of 1.00-M sodium acetate was added to MXC-1 anodic chambers to give an initial concentration of 10 mM just prior to inoculation, and concentrations were kept near this value until around day 45. From this time on, the amount added periodically was reduced to maintain concentrations at 1–2 mM because this was sufficient to yield maximum current production, and closer to conditions in the natural environment.

The potential of graphite working electrodes was controlled using a potentiostat (VersaStat MC, PAR/Ametek, Oak Ridge, TN, United States). The startup potential was +10 mV (all values vs. SHE), but changed to a highly electropositive value of +410 mV after 7 days without any increase in current, the rationale being to provide a highly favorable thermodynamic situation for electroactive bacteria in the sediment. This electropositive potential was maintained for 3 months, at which time the potential was dropped to −190 mV, a value that allowed ca. 25% of the maximum current production as measured by current-voltage and cyclic voltammetry experiments (Supplementary Figure S4B), and also represents the lower range of natural iron-oxide mineral reduction (Thamdrup, 2000). MXC-1 was maintained in a fed-batch manner for almost 4 years.

At ∼9.5 months of operation of MXC-1, a second cell, MXC-1.1, was prepared (see Supplementary Methods for details) and inoculated with planktonic medium taken directly from MXC-1 anodic compartments (a mixture of 10 mL from each of the two parent chambers was added to each daughter chamber). The main aim of this second MXC was to assess whether different EET-active communities, species, or even strains would be enriched compared to MXC-1, and whether they would be most abundant in the planktonic medium or as part of anode-biofilm consortia. The startup potential imposed at the graphite working electrodes was −190 mV. As with MXC-1, acetate was added periodically from a 1.00-M stock solution to give final concentrations of 1–2 mM.

Both MXCs were operated at ambient room temperature. Temperature was recorded at 5-min intervals with a data logger (HOBO Pendant, Onset, Bourne, MA, United States), and ranged from 18 to 24°C. Current readings were taken at 5-min intervals for the entire experiment, except when performing voltammetry or other electrochemical measurements. Anode and cathode media were exchanged at intervals ranging from 2 to 6 weeks, leaving at least ∼10% of the spent medium during each exchange. The maintenance anode potential for MXC-1 was −190 mV for most of the study. For MXC-1.1, the potential was −190 mV for the first 6.5 months, and was set to increasingly negative values over time (−220 to −250 mV) in order to maintain similar levels of steady-state current production. This effect could have been caused by selection for more efficient EET-active organisms over time, but is also confounded with possible drift in the reference electrode voltage leading to increasing systematic error.

### Biomass Sampling and DNA Extraction

For both MXCs, planktonic samples were collected during most medium exchanges. Anode biofilms for MXC-1 were sampled at two timepoints: 10 months, and near the end of the study (39 months). The first biofilm samples were characterized using 16S rRNA gene clone libraries, while the final biofilm samples (two different patches from a single anode) were included in our metagenomic analysis, and therefore are the key samples of this study. See Supplementary Figure S2 for an experiment overview and timeline. Details for all sampling events follow.

Biomass for preliminary 16S rRNA gene clone library analysis of the MXC-1 planktonic community in the Anode 1 (A1) chamber was collected at 4 months post-inoculation from 0.2-μm filters through which 30 mL of A1 medium had been passed. DNA was extracted, quantified and amplified by PCR on the same day (details below). Samples of the anode-attached biofilms of this MXC were collected after 10 months of continuous operation with electrodes poised mostly at −190 mV. Each of the graphite electrodes from A1 and A2 were briefly removed and exposed to ambient air while sampling, then reinserted to allow biofilms to recolonize anode surfaces. Sterilized razor blades were used to gently remove most of the biomass from the graphite surface, and biomass was then flushed into 2-mL centrifuge tubes using a small amount of sterile anaerobic media. Samples were stored at −80°C within 30 min. Details of genomic DNA isolation, PCR amplification, sequencing and phylogenetic analysis are given in the Supplementary Methods (the initial 16S rRNA gene sequencing analysis is not the main focus of this manuscript).

To explore the planktonic community composition and its dynamics across time in the daughter reactor MXC-1.1, we applied metagenomic sequencing to samples collected at a variety of timepoints within a few months of inoculation (five samples from 2 to 5 months post-inoculation) and near the end of the experiment (four samples from 34 to 36 months). We also included planktonic samples from the parent reactor MXC-1 near the end of the experiment for comparison (Supplementary Figure S2). Details relating to DNA extraction and sequencing are provided in Supplementary Table S1.

Samples selected for metagenomic analysis were collected and processed as follows. For planktonic samples (*n* = 26, collected from both MXC-1 and -1.1), 40–45 mL of spent anode medium was collected in centrifuge tubes and spun for 15 min at 5,000 × *g* to pellet cells. The supernatant was discarded and cell pellets were stored at −80°C until DNA extraction. At 39 months, biofilm samples (*n* = 2, replicate samples from MXC-1, A2) were collected as described above for the clone library. For all samples, DNA was extracted using a PowerBiofilm DNA Isolation Kit (MO BIO Laboratories, Inc., Carlsbad, CA, United States) and concentrations were quantified using a Qubit dsDNA High Sensitivity Assay Kit and 2.0 Fluorometer (Invitrogen/Thermo Fisher Scientific).

### Metagenomic Sequencing, Assembly and Genome Binning

DNA library preparation and metagenomic sequencing were performed by the UC Davis DNA Technologies Core^1^. Samples were prepared for sequencing on the Illumina HiSeq 3000 platform using paired-end reads with an average length of 150 base pairs and target insert size of 350 base pairs. All 28 samples (26 planktonic plus two biofilm) were run in a single lane. A fluidics problem occurred during the first run, and a second identical library and sequencing run was performed. As the data from both the first and second runs were deemed to be of sufficient quality and similarity, reads from the two runs were pooled for downstream analysis.

Metagenomic reads from each of the 28 samples were processed and assembled separately following our laboratory’s standard data preparation procedure for ggKbase^2^. Briefly, forward and reverse reads were checked for Illumina adapters, PhiX, and other Illumina trace contaminants using BBTools^3^. Sequences were trimmed using Sickle^4^ and reads were assembled using IDBA_UD (Peng et al., 2012) with the ‘-pre_correction’ option. Resulting scaffolds ≥ 1 kb in length were mapped using Bowtie 2 (Langmead and Salzberg, 2012) with default settings. Prodigal (Hyatt et al., 2010) was used for gene prediction, and similarity searches of the corresponding protein sequences were run against the KEGG (Kanehisa et al., 2016), UniRef100 and UniProt (Suzek et al., 2007) databases using USEARCH (Edgar, 2010). The 16S and 23S rRNA gene sequences were identified using an in-house script that utilizes Infernal (Nawrocki and Eddy, 2013) to perform hidden Markov model (HMM) searches based on databases from the SSU-ALIGN package (Nawrocki, 2009); see Brown et al. (2015) for details. Prediction of tRNA genes was accomplished using tRNAscan-SE (Lowe and Eddy, 1997).

Each of the 28 assemblies were binned separately. Manual binning was performed using the graphical interface on ggKbase^5^, which allows the user to select and bin scaffolds based on their GC content, average read coverage, and predicted taxonomy (based on gene annotations and single copy gene inventory).

### Genome Curation and De-Replication

The script ‘ra2.py’ was used to identify and correct assembly and scaffolding errors (Brown et al., 2015). If errors could not be corrected, NNNs were inserted. In the absence of paired read support for the join, the original scaffold was split. Assessment of genome completeness was based on the presence or absence of genes deemed to be universal and present in a single copy in most bacteria or archaea (Raes et al., 2007). Genomes were considered to be draft quality if at least 70% of these single-copy genes (SCGs) were present (36/51 for bacteria and 27/38 for archaea), and near-complete if ≥90% of the SCGs were identified. Because all of the 28 samples were derived from the same primary inoculum, and many were from duplicate anodic chambers collected at the same time as part of time series, it was expected that some samples would contain largely overlapping populations; genome de-replication across all samples was thus a critical step. Draft-quality genomes (≥70% SCGs) from all samples were compared using an in-house script (Probst et al., 2017) that generates an alignment of all scaffolds in each individual genomic bin to the scaffolds of every other bin using the NUCmer extension of the MUMmer program (Delcher et al., 2002). For any sets of genomes that were at least 98% similar across 70% or more of the total alignment length (to be consistent with the 70% completeness threshold based on SCGs), a representative genome was selected based on the following criteria (in order of priority in cases of ties): (i) genomes with the highest score, where the score was calculated with the formula (number of non-redundant SCGs) – 2 × (number of multiple SCGs); (ii) the largest N50 scaffold length; (iii) total genome size (i.e., the sum of the lengths of all contigs in a bin).

### Phylogenetic Analysis

Both 16S rRNA and ribosomal protein S3 (rpS3) genes were used for phylogenetic analysis of genomes. Structure-based sequence alignments of 16S rRNA genes were generated with SSU-ALIGN^6^ (Nawrocki, 2009). A minimum aligned length of 800 bp was required for inclusion in construction of phylogenetic trees. Alignments of rpS3 sequences were performed using MUSCLE with default parameters (Edgar, 2004). Sequences less than 50% of the aligned length were excluded. Maximum-likelihood phylogenies were inferred using RAxML version 7.2.8 (Stamatakis, 2014) with either the GTRCAT (16S rRNA) or PROTCATJTT (rpS3) model of evolution and 100 bootstrap re-samplings.

### Metabolic Overview of Genomes

Genome summaries based on gene annotations to were used to predict the likely primary metabolic strategy of the organisms represented in our curated genome set (made using ggKbase). For enzymes and metabolic pathways of particular relevance in our study, for example those involved in acetate utilization and carbon fixation, annotation-based searches and all results were checked manually and the search terms iteratively refined, relying on protein BLAST searches and sequence alignments when necessary. A compilation of the terms and criteria used for all annotation-based searches can be found in Supplementary Files S1, S2. Genes annotated as ribulose 1,5-bisphosphate carboxylase/oxygenase (Rubisco) were classified by building a phylogenetic tree containing sequences from other bacterial and archaeal type strains (Supplementary File S3). Only those that clustered with form I or form II sequences, and thus likely involved in carbon fixation, were counted (Tabita et al., 2007).

### Identification of Protein-Coding Genes Implicated in Extracellular Electron Transfer

Genes encoding multiheme *c*-type cytochromes were identified by searching the translated protein sequences of all genes for Cxx(xx)CH heme-binding motifs, where C is cysteine, H is histidine, and “x” is any residue. The motif CxxCH is by far the most common, but there are known cases of larger motifs (Sharma et al., 2010), and we therefore allowed for 2–4 variable “x” residues in the search. Proteins were categorized as MHCs if they contained three or more motifs. Subcellular localization of cytochromes with three or more heme-binding motifs was predicted using PSORTb version 3.0.2 (Yu et al., 2010).

Putative outer-membrane porin proteins that could be involved in extracellular electron transfer as part of porin-cytochrome complexes (PCCs) were identified using hidden Markov models (HMMs) (Eddy, 2011). Four separate models were constructed based on the following porin proteins from *Shewanella oneidensis* MR-1 and *Geobacter sulfurreducens* PCA: MtrB (SO_1776) (Hartshorne et al., 2009), OmbB (GSU2733), ExtB (GSU2644)/ExtE (GSU2726), and ExtI (GSU2939) (Liu et al., 2014; Chan et al., 2016). For each of the four groups above, protein BLAST searches (Altschul et al., 1990) against the NCBI non-redundant protein (nr) database (2017/03/25) were conducted to gather a set of the most closely related proteins in public databases. All hits with *E*-values less than 10^20^ to 10^30^ (minimum 26–27% identity) were retained for building the HMMs. Sequences were aligned using MAFFT v7.222 (Katoh and Standley, 2013) with default parameters. The ‘hmmbuild’ function of HMMER v3.1b2^7^ was used with default parameters to construct HMMs, which were then used to search our curated metagenomic dataset for homologs with ‘hmmsearch.’ Putative porin genes identified by the HMMs had to meet two criteria to be assigned as part of a putative PCC. First, the gene must be in proximity to at least one MHC on the metagenomic scaffold; this was assessed visually in ggKbase for each candidate gene. Second, the protein must have a minimum of 14 predicted transmembrane domains (all but two cases had 16 or more domains). The number of transmembrane beta strands for each putative porin was predicted using the web-based PRED-TMBB with the default Viterbi method^8^. For all of the putative porins meeting these requirements, protein BLAST searches were again performed as described above to gather the most closely related sequences from NCBI databases.

Potential e-pili were identified largely following the methods of Bray et al. (2020). The model and query protein sequence was PilA from *Geobacter metallireducens* GS-15 (WP_004511668.1, locus Gmet_1399) (Tan et al., 2017). Proteins from the 86 non-redundant assembled genomes were used to create a BLASTP (version 2.10.0+) database, which was then queried with the PilA sequence. All resulting sequences (*n* = 272) were analyzed using Pilfind^9^ to help verify pili sequences as type-IV (Imam et al., 2011), leaving 224 sequences. A Python script made freely available^10^ by Bray et al. (2020) was used to calculate the number of aromatic amino acids (F, W, Y, and H), the length of the mature pilin sequence, and the percentage of aromatic residues. Sequences with ≥8% aromatic residues were then manually selected by building MAFFT (v7.309) protein alignments (Katoh and Standley, 2013) in Geneious (v9.1, Biomatters) and inspection of aromatic spacing and distribution.

A search for cytochrome OmcS homologs was performed as above for e-pili against the same internal BLASTP database, using the *G. sulfurreducens* OmcS sequence (WP_119334099.1) as query, giving 47 sequences with default search parameters. As above, protein alignments were used to assess sequence homology. A length cutoff of 500 amino acids was deemed appropriate, reducing the set to 36 sequences. Further alignments and manual curation reduced the final set to 22.

## Results

### Enrichment of Electroactive Microbial Consortia From the Rifle Subsurface

Over the 3-month startup period of MXC-1, during which the electron-accepting anode was poised at a thermodynamically favorable potential of +0.4 V, thin and mostly colorless biofilms formed on the graphite anodes. After changing the anode potential to a more environmentally relevant and thermodynamically challenging potential of −0.2 V (Thamdrup, 2000), thicker red-colored biofilms formed on distinct regions of the electrodes in both chambers of MXC-1, covering approximately half of each anode (see Supplementary Figure S3A). Initial 16S rRNA gene clone library analysis of the MXC-1 planktonic community (sampled at 4 months) showed a predominance of Bacteroidetes, Betaproteobacteria, Tenericutes, and Gammaproteobacteria. Anode biofilms were sampled at 10 months (after performing other biofilm cyclic voltammetry experiments) for similar 16S rRNA gene analysis, which revealed distinct differences in community composition of the red vs. colorless biofilm regions (Supplementary Figure S3B). For biofilm sampling, the anodes were extracted from the MFC, sampled to remove sufficient biomass, and reinserted. Despite biomass loss and brief exposure to aerobic conditions, steady-state current production recovered within 1 day and the CV signatures were unaltered. The two red biofilm samples were dominated by bacteria related to *Geobacter* and *Pelobacter* spp. (82 and 84% of clones for anodes 1 and 2, respectively) whereas the colorless biofilm contained a more even distribution of taxa similar in composition to that of the 4-month planktonic 16S rRNA library. After the switch to −0.2 V at 3 months and subsequent growth of the red biofilm, maximum current production in MXC-1 approached values reported for other *Geobacter*-dominated anode biofilms, ∼80 μA/cm^2^ based on geometric surface area (Supplementary Figure S4A) (e.g., Marsili et al., 2008; Torres et al., 2009). Over time the maximum achievable current decreased, likely due to membrane fouling and buildup of extracellular material on the anodes.

At 9 months, planktonic samples from each of the MXC-1 anodic chambers were mixed and used to inoculate a second bioreactor, MXC-1.1. Acetate again served as the electron donor, and anodes were poised at potentials of −0.19 to −0.24 V to limit current production and place a stronger selection pressure for organisms likely specialized in EET to, e.g., iron(III)-oxyhydr(oxide) minerals, which have a redox potential in this range. Because of the lack of visible anode biofilms in MFC 1.1 and the fact that exchange of the anode medium caused current to drop to near zero, but recovered within 3–5 h (Supplementary Figure S5), we conclude that current in MFC 1.1 was dominated by planktonic EET-active organisms, possibly utilizing soluble redox shuttles.

### Genomic Resolution of Anode-Biofilm and Planktonic MXC Communities

A total of 26 planktonic plus two duplicate biofilm samples were selected for metagenomic sequencing and analysis (see Supplementary Figure S2 for sample timeline). We reconstructed 616 draft bacterial and two archaeal genomes from the 28 independently assembled metagenomes using a combination of GC content, phylogenetic profile, coverage and single copy gene inventory. In addition, 13 plasmid and seven phage bins were also generated. Overall, 449 of the bacterial and archaeal bins were considered to be near complete (≥90% of single-copy genes, SCGs) with low levels of contamination, and 57 more were of lower quality (between 70% and 90% SCGs). After sequence curation (see section “Materials and Methods”), genome bins were de-replicated, resulting in high quality draft genomes for 84 bacterial and 2 archaeal populations, 70 of which are considered near-complete (see Supplementary File S4 for these and other genome stats and features). These genomes accounted for between 70 and 91% of the reads from each sample, averaging 81.3%. One abundant and persistent Micrococcales in the planktonic fractions of both MXCs, represented by the curated genome version “S3_Micrococcales_67_491,” was assembled as single contigs in three different samples. The 2.72-Mbp chromosome was manually curated into a single DNA fragment. An overview of the predicted metabolic capacities of all curated genomes is provided in Supplementary File S5.

Given that around two out of three bins lacked 16S rRNA sequences, which do not assemble well in metagenomic reconstructions (Miller et al., 2011), genomes were assigned to organisms based on phylogenetic analysis of conserved (e.g., ribosomal) proteins. The samples most relevant in this study are those from MXC-1, for which we sampled the biofilm of the more active bioanode near the end of the study. However, we include the genome-level community composition of all samples from both MXC-1 and -1.1 in Supplementary Figure S6, and we leveraged the metagenomic data from all samples in our analyses.

**FIGURE 1:**
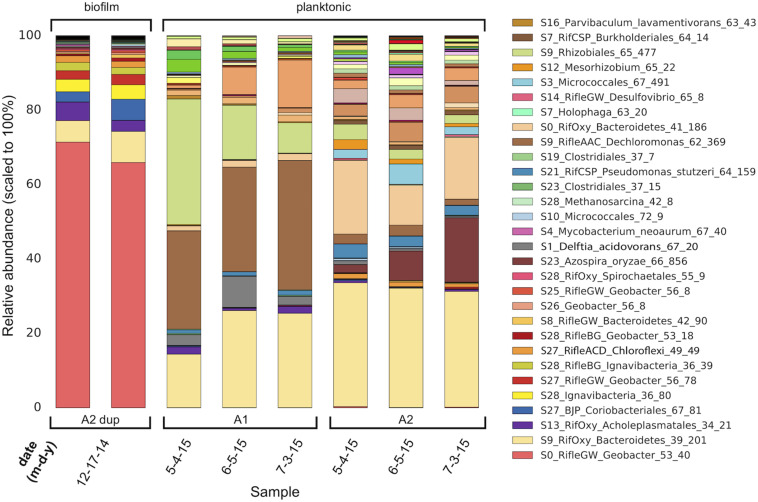
Genome-resolved overview of the composition of MXC-1 consortia across a 6-month timespan. There are major differences in the composition of Anode-2 biofilm (two semi-replicates, “A2 dup,” left columns) and planktonic samples. The planktonic consortia within each chamber (A1 and A2) of MXC-1 remain fairly constant in composition over the 2-month period covered by the samples, although the abundances of certain genomes differ between them. Genome order in each bar is according to biofilm abundance, with the most abundant at the bottom. The legend lists the 30 most abundant MXC-1 genomes in same order as in the bars.

**FIGURE 2:**
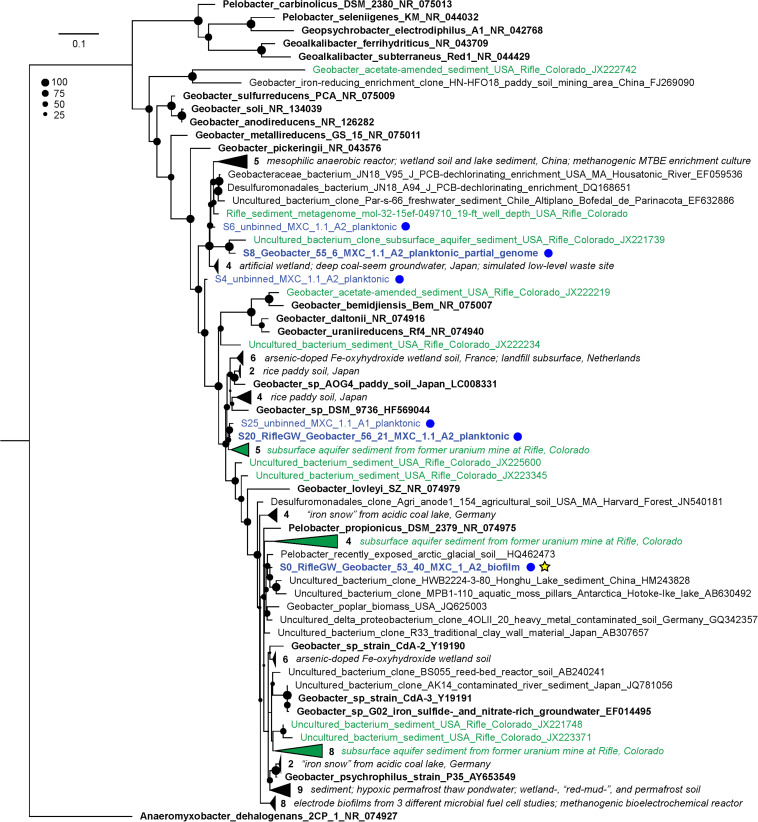
Maximum-likelihood 16S rRNA gene phylogenetic tree showing the placement of all recovered MXC *Geobacter*/*Pelobacter* sequences. MXC sequences are colored blue and also designated with blue dots at right; sequences that were assigned to a genome bin appear in bold. The genome “S0_RifleGW_Geobacter_53_40” (yellow star) made up on average 69% of binned reads in the duplicate biofilm samples, and places somewhere in the *Geobacter–Pelobacter* “subsurface clade 2” (Holmes et al., 2007). The tree includes all sequences from the NCBI non-redundant nucleotide database that have ≥97% identity to any MXC sequence. Sequences from representative isolates are shown in bold black text, and those derived from Rifle samples are colored green. Groups of closely related sequences are collapsed into wedges, with the number in each group indicated. A short descriptor of the isolation source(s) for each organism is included in the sequence name, or in italicized text to the right of collapsed clades; accession numbers are provided as the last field of each sequence name. The size of black circles at each node indicates bootstrap value (see legend).

The compositions of the duplicate MXC-1, Anode-2 (“A2 dup”) biofilm samples were similar, and distinct from that of any planktonic sample (Figure 1), in line with prior 16S rRNA gene clone library results (Supplementary Figure S3). Six near-complete and one partial *Geobacter* genomes made up on average 73% of biofilm DNA, dominated by S0_RifleGW_Geobacter_53_40, which comprised at least 69% of the total electrode-associated community. Genes for 16S rRNA were recovered in two of the seven *Geobacter* genomes, and three additional sequences from scaffolds not assigned to bins (Figure 2). Comparing these sequences with those from the original biofilm clone libraries sampled more than 2 years earlier showed that the dominant anode-attached *Geobacter* from the late-2014 metagenomic samples, or a closely related species/strain, was present in the clone libraries. The other five metagenomic *Geobacter* 16S rRNA sequences were not represented by clones (Supplementary Figure S7).

Also among the top 10 most abundant biofilm organisms were two novel Bacteroidetes, an Acholeplasmatales (Tenericute), a Coriobacteriales (Actinobacteria), two Ignavibacteria, and one Chloroflexi. The 18th most-abundant organism in the biofilm (0.26% of mapped reads) was an archaeon whose 16S rRNA gene shares 99.8% identity with that from *Methanosarcina subterranea*. *Methanosarcina* spp. are known to form EET-based syntrophic partnerships with *Geobacter* spp. (Kato et al., 2012; Rotaru et al., 2014) and sulfate-reducing bacteria (Stams and Plugge, 2009; McGlynn et al., 2015), and have shown to play a role in metal reduction at the Rifle site (Holmes et al., 2018).

### Genomes Rich in Multiheme Cytochromes and/or Putative e-Pili

Given our main objective of identifying potentially electroactive bacteria, we analyzed genomes containing many (or very large) multiheme *c*-type cytochromes (MHCs), which are fundamental in most known EET pathways (Shi et al., 2009, 2016; Wrighton et al., 2011). We also searched genomes for putative type-IV “e-pili” known to be involved in EET in *Geobacter* and likely in other taxa (Walker et al., 2018, 2020; Bray et al., 2020). Table 1 includes the 31 genomes found to have such genes. *Geobacter* species harbored the greatest numbers of MHCs, ranging from 16 to 81 with an average of 64. The most abundant of these, S0_RifleGW_Geobacter, had 72 MHCs, the largest of which contained 45 putative heme-binding sites. This genome contains the two key subunits I and II of cytochrome *c* oxidase complex IV (as does one other *Geobacter* genome, S28_RifleBG), and is thus predicted to be capable of oxygen respiration or at least able to tolerate low levels of O_2_ (Supplementary Files S5, S6). Five of the six *Geobacter* species were substantially more abundant in the biofilm than in any planktonic sample. A majority of the genomes in Table 1 also encoded putative e-pilin genes, described in more detail in Section “e-Pili and Multiheme Cytochrome OmcS” below.

**TABLE 1:** Genomes enriched in multiheme cytochromes (MHCs) and potential e-pili.

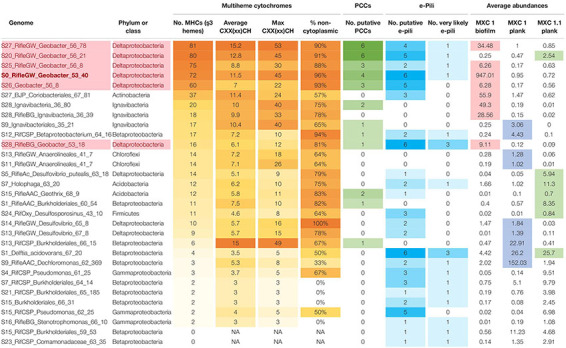

**Genomes are listed in order of the total number of putative MHCs encoded in their genomes (max 81). Genome names are in the form of “Sample-number_organism-classification_GC_Cov, where organism classification was established to the most specific level possible and, if applicable, includes a descriptor of the type of Rifle metagenome sample for which a closely related genome was found (see Table 4), GC is the %GC content of the genome and Cov is the DNA read coverage in the sample from which the representative genome was recovered. Orange, green, and blue shading scales with the numerical values in each individual column. Proteins were considered to be putative MHCs if their sequences contained at least three heme-binding motifs, CXX(xx)CH. The average number of motifs, as well as the maximum number found in a single protein-coding sequence, are given in the next orange-gold shaded columns. The number of putative porin-cytochrome complexes (“PCCs”) is also listed for each genome (green shading). The first “e-Pili” column includes 86 selected sequences with a high density of aromatic amino acids and no large gaps between them, while the second column includes only those meeting stricter criteria (see text). “Average abundances” refers to the average value for a genome’s normalized relative abundance across each of the three MXC sample types: the MXC-1 biofilm (n = 2, duplicate samples) and planktonic (n = 6), and MXC-1.1 planktonic (n = 18). Color shading across these three columns indicates the sample type for which each genome was most abundant, on average.**

Other organisms outside of the Deltaproteobacteria that were abundant in the biofilm and harbored many MHCs include a Coriobacteriales (Actinobacteria; 37 MHCs) and two taxonomically distant Ignavibacteria, whose genomes encode 18 and 20 MHCs, the largest of which, respectively, contain 40 and 33 heme-binding motifs. The results reveal a strong positive correlation between genomic MHC content and anode biofilm abundance (compare genomic MHC content with abundance values in Table 1).

Organisms detected at high abundance in the planktonic fraction with genes encoding MHCs include, from MXC-1: a third Ignavibacteria (S9, which has an identical 16S rRNA sequence with S28_36_80), two Betaproteobacteria including a Burkholderiales with a 49-heme cytochrome, two high-similarity Chloroflexi strains (order Anaerolineales), and a Deltaproteobacteria from genus *Desulfovibrio*. From MXC-1.1: two Acidobacteria from genera *Geothrix* and *Holophaga*, a Burkholderiales sp. with 11 MHCs, a *Geobacter* sp., and a novel Firmicutes (genus *Desulfosporosinus*), also with 11 MHCs.

#### Acetate Metabolism

Among the genomes with EET-related genes (Table 1), *Geobacter* species have notably more acetate transporters on average (3.0 genes annotated as such vs. 0.6 for all others). However, there is variation within the *Geobacter*, with no acetate transporter genes detected in 2/6 genomes. An extended version of Table 1 containing this and other information on the presence/absence and copy numbers of selected genes involved in key metabolic pathways can be found in Supplementary File S6. Another signature of these *Geobacter* genomes is their lack of acetyl-CoA transferase, instead possessing acetate kinase and phosphotransacetylase, as well as pyruvate ferredoxin oxidoreductase (PFOR), pyruvate dehydrogenase and pyruvate carboxylase as enzymatic routes to activate and utilize acetate in central metabolism and as a carbon source (Mahadevan et al., 2006).

Based on this metabolic summary, we conclude that 23/31 organisms represented by the genomes in Table 1 are likely able to use acetate as both an energy and carbon source (including the six *Geobacter*). Among the exceptions are two of the three Ignavibacteria (both “S28”), which appear to lack PFOR, whereas the third (S9) has five genes annotated as such, similar to *Geobacter* spp. This genome also has the two enzymes making up the glyoxylate shunt, suggesting that the S9 organism can sustain growth using only acetate-derived carbon whereas the other two cannot. However, it is possible that some of these genomes, including the two Chloroflexi, have a complete Wood–Ljungdahl pathway despite that fact that no genes annotated as acetyl-CoA synthase were found (each of these genomes did harbor all other key genes in the WLP, including 9–11 annotated as CO dehydrogenase), and can assimilate acetate-derived carbon in this manner.

#### Subcellular Localization of MHCs

The predicted location of MHCs within the cell gives clues to function, especially when combined with whole-genome analysis that often reveals the genomic position of MHC genes. This information is particularly relevant for cytochromes involved in EET, as at least some of them must be exported to the extracellular space. Results of the subcellular localizations predicted using PSORTb are summarized in Table 2. *Geobacter* spp. have a much higher percentage of MHCs predicted to be extracellular compared to all other organisms (21% vs. 5%), as well more “unknown” (48% vs. 38%), which can indicate that a protein has multiple localizations. One reason for this is that *Geobacter* genomes contain not only the most MHCs, but also those with the greatest number of heme-binding motifs, and these were predicted to only be localized extracellularly or had unknown localization. Table 1 includes the percentage of MHCs with predicted localization other than the cytoplasm or cytoplasmic membrane, which averaged 90% for *Geobacter* and 58% for all others.

**TABLE 2:** Subcellular localization of all MHCs from all non-redundant draft genomes.

	**Predicted subcellular localization of MHCs**					
**Group/genome**	**Cytoplasm**	**Cytoplasmic membrane**	**Extracellular**	**Periplasm**	**Unknown**	**Total num.**
All *Geobacter* spp.	5%	4%	21%	22%	48%	384
All others	10%	22%	5%	25%	38%	302
S0_RifleGW_*Geobacter*_53_40	1%	3%	22%	18%	56%	72

MHCs with 3–14 hemes	10%	12%	14%	21%	43%	582
MHCs with 15–27 hemes	13%	0%	20%	26%	41%	66
MHCs with 30–53 hemes	0%	0%	31%	0%	69%	38

**The freely available program PSORTb (version 3.0.2) was used to make predictions. Multiheme cytochromes are divided into different subsets (leftmost column) to highlight observed trends. The upper half of the table is grouped by genomes, and the lower half by the number of CxxCH heme-binding motifs in putative MHC sequences. The rightmost column gives the total number of MHCs in each subset*.*

Signal peptides are another indicator that a protein is likely exported. For all MHCs taken together (*n* = 686), 45% have a signal peptide. However, if they are disaggregated into a smaller and larger subset (in terms of the number of heme-binding sites), only 36% of those with 3–20 motifs have a signal peptide, in contrast to 72% for those with >20 motifs. The complete dataset and analyses for PSORTb results can be found in Supplementary File S7.

#### Porin-Cytochrome Complexes

**FIGURE 3:**
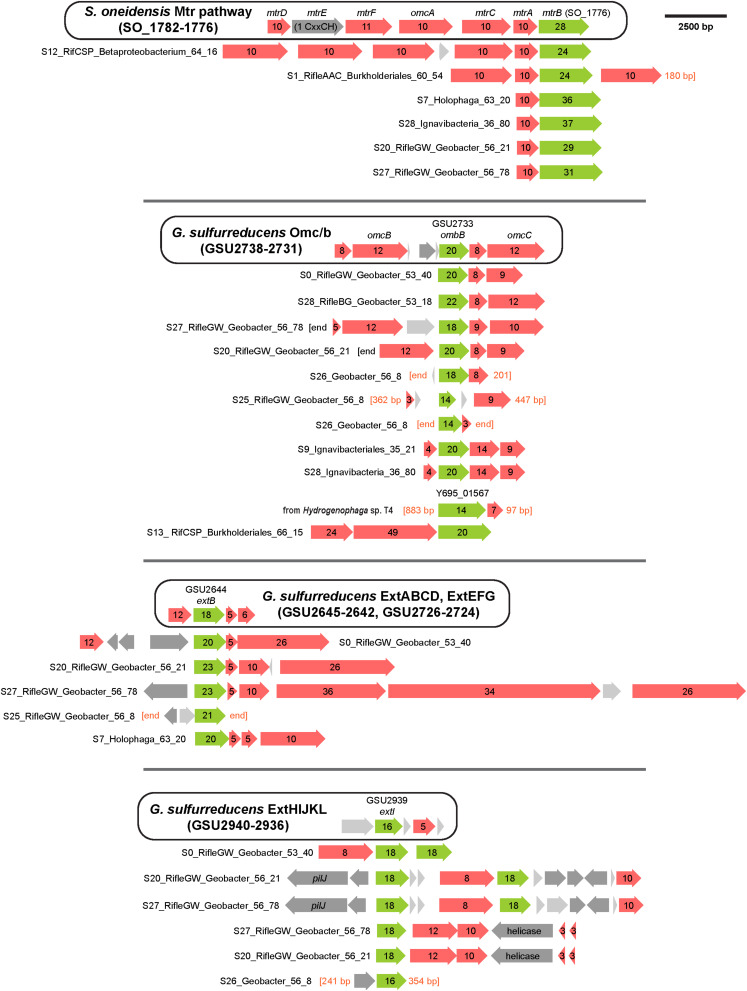
Porin-cytochrome gene clusters found in MXC genomes. Hidden Markov models based on known porin proteins from the type species *G. sulfurreducens* and *S. oneidensis* were used to identify putative cytochrome-associated porin proteins in the MXC metagenome. Four distinct porin families from these model organisms were used as inputs, and the results above are grouped according to family type. The canonical porin-cytochrome gene cluster from *Shewanella* or *Geobacter* is shown at the top of each group for comparison. Green arrows represent porin proteins (numbers indicate predicted transmembrane domains), red arrows indicate multiheme *c*-type cytochromes (numbers indicate heme-binding motifs), dark gray arrows indicate other genes (pilJ is a pilus gene), and light gray arrows indicate proteins with no predicted function.

To support the inference of EET capability based on genomic MHC content, we searched MHC-encoding regions for putative outer-membrane porin proteins. Together with MHCs, porins have been experimentally shown to form membrane conduits for electrons called porin-cytochrome complexes, PCCs (Liu et al., 2014). We used hidden Markov models based on known porin proteins to search MXC genomes; see Section “Materials and Methods” for details. Twelve of the 53 near-complete genomes that contain one or more MHCs harbor at least one putative PCC; all 12 are among the organisms whose genomes encode the greatest number of MHCs (Table 1). Protein sequence alignments and phylogenetic trees for the putative porins we identified suggest three or four evolutionarily independent families (Supplementary Figure S8) (cf. Shi et al., 2014; Chan et al., 2016). Gene diagrams for all putative PCCs from this study, as well as *Geobacter* and *Shewanella* type-strain examples, are grouped by porin family type in Figure 3, and summary statistics are listed in Table 3.

**TABLE 3:** Summary statistics for putative porin cytochrome complexes (PCCs) (shown visually in Figure 3).

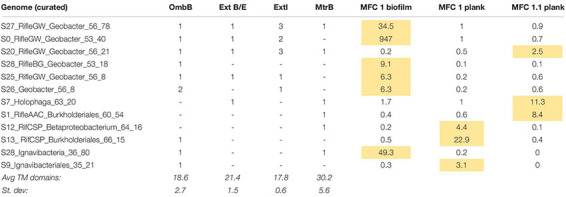

**Twelve of the 86 near-complete genomes contain at least one putative complex with homology to currently known outer-membrane PCC porin protein families from Gram-negative bacteria (Liu et al., 2014; Chan et al., 2016). The number of putative porins of each family type are listed by genome, along with the average and standard deviation of the number of beta-strand transmembrane domains predicted by PRED-TMBB (bottom rows). The average relative (rel.) abundances of each genome in MXC-1 biofilm, planktonic (plank.), and MXC-1.1 planktonic samples are also given for reference, with tan shading indicating the sample type for which abundance was highest on average.**

*Geobacter* spp. had the greatest number and diversity of porin types, including proteins homologous to MtrB from *Shewanella* spp., which have also previously been identified in several other phyla (Shi et al., 2014). More notably, putative PCCs were also identified in an Acidobacteria (genus *Holophaga*), three Betaproteobacteria (two of order Burkholderiales), and two Ignavibacteria. The *Holophaga* S7 genome contains two PCCs, one sharing homology with MtrB, the other in the ExtB/E family. To our knowledge, this is the first report of a likely PCC in Acidobacteria.

Both Ignavibacteria genomes encode OmbB-like PCCs, and one of them (S28) also contains a close MtrB homolog. *Geobacter*-like PCCs have been previously identified in the two Ignavibacteria type strains *Melioribacter roseus* and *Ignavibacterium album* (Podosokorskaya et al., 2013; Shi et al., 2014), and in metagenomics-derived environmental genomes from an iron-rich hot spring (Fortney et al., 2016). However, to our knowledge this is the first report of a PCC porin with homology to MtrB from this group.

Porin-cytochrome complexes with porins homologous to MtrB have been found in a diversity of Betaproteobacteria, including Fe(II) oxidizers such as *Sideroxydans lithotrophicus*, and Fe(III)-reducing species like *Rhodoferax ferrireducens* (Shi et al., 2012; He et al., 2017). In addition to Mtr homologs, we found one case of a PCC similar to OmbB in the genome Burkholderiales S13. The porin is preceded by two large MHCs containing 24 and 49 heme-binding motifs. A protein BLAST against the NCBI database revealed a highly related porin (Y695_01567) in the isolate genome of *Hydrogenophaga* sp. T4 (AZSO01000162), which is adjacent to a MHC with seven heme-binding motifs. As this is the only other open-reading frame on the short scaffold containing these two genes, it is possible that other proximal MHCs form part of a PCC in sp. T4.

**FIGURE 4:**
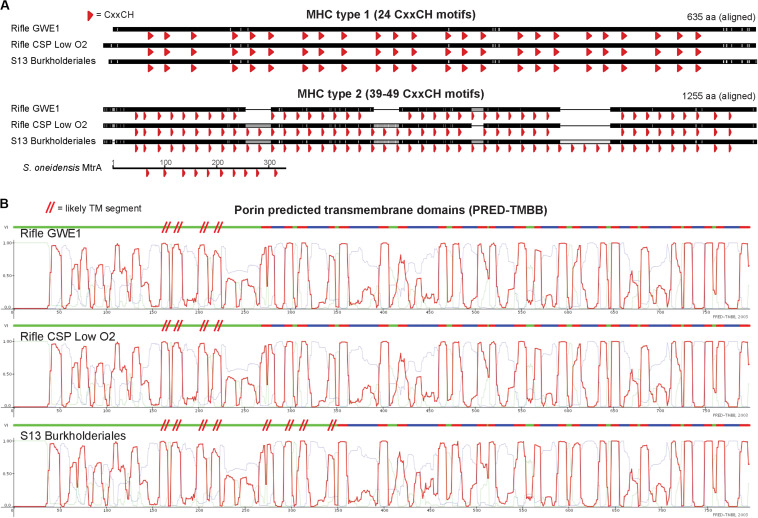
Comparison of homologous MHC and porin protein sequences from three closely related Burkholderiales genomes. **(A)** Protein alignments of MHCs showing heme-binding motifs as red arrowheads emphasize the dense packing of hemes, as well as the variability in number of heme-binding motifs for the larger (“type 2”) proteins. The decaheme cytochrome MtrA from *Shewanella oneidensis* MR-1 is shown using the same horizontal scale (amino-acid sequence length) as the three larger MHCs. **(B)** Despite high porin protein sequence similarity between the S13 and Rifle Burkholderiales genomes (97.6 and 97.7%), PRED-TMBB with default parameters predicted 20 vs. 24 transmembrane domains (indicated by red-colored segments above each profile). We therefore added red parallel hashes to indicate likely additional transmembrane regions based on visual inspection. Light green and blue traces correspond to predicted cytoplasmic or non-cytoplasmic localization.

The porin sequence from Burkholderiales S13 is most similar to those from two Rifle Burkholderiales genomes (Anantharaman et al., 2016), which also contain the two large MHCs preceding the porin (see Figure 3). The porins and the smaller of the two adjacent MHCs share 96–98% sequence identity, whereas the larger MHCs are less similar (80–89%) due to apparent insertion or deletion of CxxCH-containing domains. The alignments shown in Figure 4 emphasize these similarities and differences. He et al. (2017) identified similar PCCs in a number of Alpha-, Beta-, and Gammaproteobacteria that may share homology. These MHCs display a remarkably high density of heme-binding motifs: the 24- and 49-heme cytochromes use only 22.4 and 23.4 residues per heme, respectively (for reference, the decaheme periplasmic cytochrome MtrA from *Shewanella oneidensis* MR-1 has 25.1 residues per heme; see Figure 4). This suggests that these proteins might function as “molecular wires” in EET (He et al., 2017) and/or as biological capacitors for charge storage (Esteve-Núñez et al., 2008).

**FIGURE 5:**
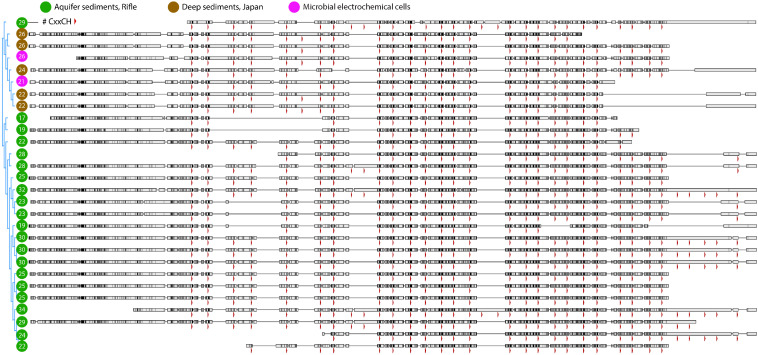
Protein alignment of large multiheme cytochromes found in closely related Chloroflexi genomes. The most closely related multiheme cytochromes to those from the MXC Anaerolineales genomes (magenta circles) were from organisms found in deep borehole sediments from Japan (brown circles), and next from Rifle, CO, United States sediment (green circles). As in Figure 4, the positions of heme-binding motifs are indicated by red arrowheads. The total number of motifs in each sequence is listed within the circle at left. Note that there are few cases where the most closely related sequences differ by only a single heme in a certain position, which may indicate that CxxCH motifs are more likely gained or lost in pairs or multiples during single evolutionary events.

We did not identify PCCs in the Anaerolineales (Chloroflexi) genomes that also encode several large MHCs, probably because they do not have a Gram-negative cell envelope (Sutcliffe, 2011; White et al., 2016). Similar to the Burkholderiales sequences described above, the largest MHCs in the two Anaerolineales genomes vary in the number of heme-binding motifs. Given that the number and positioning of hemes should impact the redox properties of these proteins, we searched for closely related sequences from public databases and from the Rifle metagenome to gain a more detailed view of this phenomenon. A manually curated alignment of these sequences is shown in Figure 5.

#### e-Pili and Multiheme Cytochrome OmcS

**FIGURE 6:**
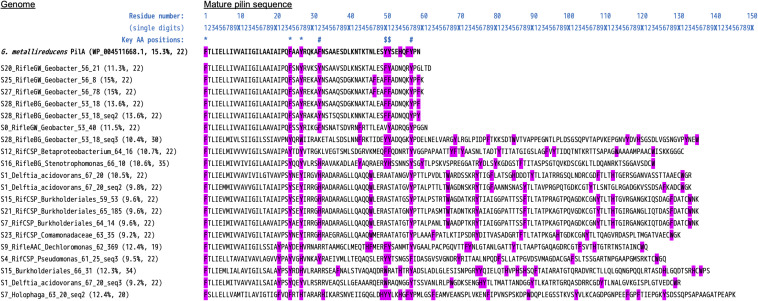
Alignment of type-IV pilin protein sequences highly similar to PilA from *Geobacter metallireducens*. Included here are the 20 sequences (from 16 unique genomes) that meet the strict criteria of Bray et al. (2020) to be considered as likely conductive “e-pili.” Namely, aromatic residues at positions 1, 24, 27, 50 and/or 51, and 32 and/or 57. The query/reference sequence we used in conducting our search is also shown (top sequence, bold text). The N-terminal pre-pilin regions have been removed, leaving the “mature” sequences. The values in blue text above are a ruler to help determine residue numbers, and symbols mark the positions of the key residues listed above [based on studies of *G. sulfurreducens*; see Walker et al. (2018, 2020)]. Two values are listed in brackets after each “Genome_(SequenceNumber)”: The percentage of aromatic residues, and the length of the maximum gap between aromatic residues.

From the initial BLASTP results obtained by searching all MXC genomes for sequences similar to PilA from *G. metallireducens*, a total of 86 pilin sequences were retained as putative e-pili (Supplementary File S8). Among the genomes with EET-related genes, 16/31 contained pili meeting the strict criteria used by Bray et al. (2020): ≥9.8% aromatic amino acids (at least between residues 1 and 59 of the mature pilin sequence), no gap between aromatic residues greater than 22, and aromatic residues at positions 1, 24, 27, 50 and/or 51, and 32 and/or 57 that were found to be essential for conductivity in *G. sulfurreducens* e-pili by Walker et al. (2018, 2020) (Table 1). This includes 5 out of 6 *Geobacter* genomes, as well as S9 Ignavibacteriales, S7 *Holophaga* (Acidobacteria) and a number of Beta- and Gamma-proteobacteria (S1 *Delftia acidovorans*, S9 *Dechloromonas*, S4 *Pseudomonas*, five Burkholderiales genomes, and S16 *Stenotrophomonas*). An alignment of these mature pilin protein sequences (*n* = 20) is shown in Figure 6.

With less stringent criteria that allow for some variation in aromatic residue positions, or consider different sequence regions that have an equally high aromatics density for longer sequences, 24/31 genomes would be included in the set with putative e-pili. An expanded version of Figure 6 containing all 86 putative e-pilin sequences is provided in Supplementary File S9. These results are consistent with recent findings from the studies cited above, and also expand the diversity of bacteria found to harbor potential e-pilin genes.

Among *Geobacter* genomes, the one harboring the most putative e-pilin genes (S28 RifleBG) was most closely related to organisms detected from Rifle background sediment (i.e., sediment that had not been amended with acetate). A notable correlation is that this genome also contains the fewest MHCs (16 compared to an average of ∼74 for the other 5 *Geobacter*), and was the only one lacking any cytochrome OmcS homologs. We searched all genomes specifically for homologs to cytochrome OmcS in light of recent evidence that this protein self-assembles to form conductive filaments in *G. sulfurreducens*, which along with e-pili have been described as bacterial “nanowires” (Filman et al., 2019; Wang et al., 2019). A total of 22 sequences were deemed to be true OmcS homologs, with all but one of these found in five of the six *Geobacter* genomes (see Supplementary Table S2 and Supplementary File S6). The one non-*Geobacter* sequence from the actinobacterial genome S27 BJP Coriobacteriales shared only 28% amino-acid identity, but protein alignment reveals clear sequence homology, including all six CxxCH heme-binding motifs (Supplementary File S10).

### Linking MXC Genomes to Rifle and Other Environments

To evaluate whether organisms identified in these MXC experiments are relevant in microbial communities present in the Rifle aquifer, we compared rpS3 and 16S rRNA gene sequences to those from 35 independent Rifle metagenomes previously assembled. The Rifle comparison dataset contained a total of 17,056 rpS3 sequences (minimum 50 residues) and 5,598 16S rRNA gene sequences. A maximum-likelihood phylogenetic tree containing all MXC sequences with those from closely related Rifle genomes is given in Supplementary Figure S12.

Thirty-eight of the 86 MXC genomes contain rps3 protein sequences sharing at least 96% identity with one or more genomes assembled from Rifle metagenomic data (Table 4), and 25 of these genomes are ≥99% similar to one or more of 47 unique Rifle genomes (Supplementary Table S3). A majority of these closely related Rifle genomes were derived from acetate-amended groundwater samples, followed by acetate-amended sediment and naturally low-oxygen groundwater (see Table 4).

**TABLE 4:** MXC genomes that are closely related to those assembled from other Rifle, Colorado sediment and groundwater metagenomes.

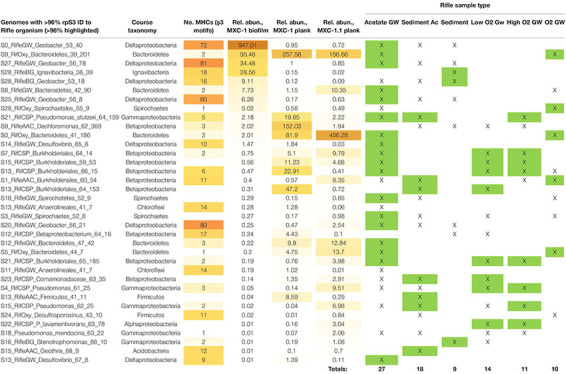

**Genomes are listed if their ribosomal protein S3 (rpS3) sequence shares ≥96% amino acid identity (ID) with a sequence from a genome assembled from the Rifle-site metagenomes (the site from which the sediment inoculum used to inoculate MXC-1 was collected). This list includes 38 of the total 86 MXC genomes, which are sorted by relative abundance (Rel. abun.) in the biofilm samples (MXC-1, Anode 2). Rifle metagenomes have been obtained from acetate (Ac)-stimulated sediment and groundwater (GW), from groundwater with naturally low (Low O_2_ Gw) and high (High O_2_ GW) oxygen concentrations, and from an oxygen injection experiment (O_2_ GW). An “X” indicates that an MXC genome is represented in that Rifle metagenomic sample. Green-shaded boxes indicate that a rpS3 protein sequence shares at least 98% identity with the corresponding Rifle-genome sequence. The total number of MXC genomes found in each sample type is shown below each column. Also shown for each genome are the number of multiheme c-type cytochromes present and average relative abundance in MXC biofilm or planktonic samples*.*

Phylogenetic trees were constructed to further place MXC organisms of interest in context with known isolates and uncultured microbes previously detected at Rifle and in other environments. The most abundant biofilm organism, S0_RifleGW_Geobacter_53_40, falls within the *Geobacter*/*Pelobacter* clade (Holmes et al., 2007) according to both 16S rRNA and rpS3 gene sequence identity (Figure 2 and Supplementary Figure S12). This organism shares 97.7% 16S rRNA gene nucleotide identity with that of *Pelobacter propionicus* DSM 2379, and 100% rpS3 amino-acid identity with sequences from several Rifle genomes assembled from acetate-amended groundwater samples. Two additional MXC *Geobacter* strains, S27 and S20 (with 81 and 80 total MHCs, the most of any genomes in this study), also share 100% rpS3 amino-acid identity with a *Geobacter* genome assembled from acetate-amended groundwater at Rifle. Strain S27 was more abundant in the anode biofilm than in planktonic samples, whereas S20 showed the opposite trend. A third abundant biofilm *Geobacter*, S28, also shares 99.5% rpS3 sequence identity with one found in Rifle sediment collected 19 ft below the ground surface and not subjected to acetate amendment. Thus, we conclude that several of the *Geobacter/Pelobacter* organisms studied here could be important players in mineral redox transformations at the Rifle site.

The fourth most abundant anode biofilm genome, the Actinobacteria S27 Coriobacteriales with 37 MHCs, had no close known relative in the Rifle metagenome. Instead, the most closely related sequences (97–98% 16S rRNA gene identity) came from uncultured organisms detected in (deep) sediment or groundwater samples in redox zones associated with iron reduction (Kimura et al., 2010) and from the biocathode of a microbial electrolysis cell (Croese et al., 2011) (Supplementary Figure S9). The two MXC Chloroflexi genomes with large MHCs also share ≥96% rpS3 identity with Rifle genomes (Supplementary Figure S10).

Two of the three MXC Ignavibacteria genomes (S28_36_80 and S9) are not represented in any Rifle metagenomic samples, but share up to 99.8% 16S rRNA gene sequence identity to organisms found in freshwater and marine sediments as well as anaerobic bioreactors (Supplementary Figure S11). The third Ignavibacteria genome (S28_36_39) does appear to have close relatives in Rifle background sediment (98.1% rpS3 amino acid identity).

The MHC-rich genomes of three phylogenetically distinct Betaproteobacteria all have close Rifle relatives. Most of these were derived from groundwater samples, which is consistent with the fact that all three were detected at highest abundance in planktonic MXC samples. Burkholderiales S13 shares 97.6% rpS3 identity with *Hydrogenophaga taeniospiralis*, likely placing it in this genus. Burkholderiales S1 shares 92.3% rpS3 sequence identity with *Rhodoferax ferrireducens*, a known iron reducer (Finneran, 2003). Betaproteobacteria S12 is not closely related to any known organism outside of Rifle, and places in the Nitrosomonadales or Rhodocyclales orders. The two Acidobacteria from genera *Geothrix* and *Holophaga* (both encoding 12 MHCs, and *Holophaga* S7 with two putative PCCs) are closely related to genomes from acetate-amended Rifle sediment.

Finally, two archaeal *Methanosarcina* genomes were assembled from MXC-1 samples. Genome S28 was more abundant in the biofilm (Anode 2), whereas S10 was at much higher relative abundance in the Anode-2 planktonic samples. The 16S rRNA gene sequence recovered from S28 is 97.8% similar to that from a Rifle genome derived from a 19-ft-deep background sediment sample, and shares 99.1 and 99.8% identity to genes from isolates *Methanosarcina lacustris* Z-7289 and *Methanosarcina subterranea*, respectively. As noted in section “Genomic Resolution of Anode-Biofilm and Planktonic MXC Communities,” *Methanosarcina* species were recently shown to be implicated in metal reduction specifically at the Rifle site (Holmes et al., 2018).

## Discussion

The composition of biofilm and planktonic samples differed substantially, suggesting that different metabolic strategies predominate in these fractions (Figure 1 and Supplementary Figure S3B). The strong correlation between genomic MHC content and abundance in the biofilm (Table 1) underscores the importance of these proteins in growth on electrodes via EET-based respiration. Based on these findings, we identify a diverse group of bacteria that may rely solely or partially on extracellular electron acceptors for growth in sediments.

Our finding that a *Geobacter* species made up ∼70% of the biofilm population is not surprising given the many prior studies using iron oxides or electrodes as the electron acceptor (and acetate as the substrate) that showed enrichment of *Geobacter* (Lee et al., 2003; Torres et al., 2009; White et al., 2009; Bond, 2010; Jung and Regan, 2011). However, the closest relatives to this abundant MXC *Geobacter* in Rifle metagenomic datasets were all derived from acetate-amended groundwater samples, with no closely related species detected in the sediment. The true environmental niche and role of this organism therefore remains an open question.

An analysis of key genes and metabolic pathways involved in acetate catabolism and carbon fixation suggest that ∼3/4 of the 31 organisms in Table 1 can likely utilize acetate as both an energy and carbon source (Supplementary File S6). Given that the anode is the only favorable electron acceptor in the system, this suggests EET-dependent acetate respiration and growth as a strategy used by this taxonomically diverse set of organisms. Coriobacteriales S27 is to our knowledge only the second neutrophilic Actinobacteria shown to encode MHCs in its genome (including one gene homologous to OmcS from *Geobacter* spp.), the prior example being a strain from deep subsurface sediments that was genomically characterized in our lab (Hernsdorf et al., 2017). Until recently, the only Actinobacteria known to perform dissimilatory iron (or electrode) reduction were obligate acidophiles whose genomes do not encode MHCs (Bridge and Johnson, 1998; Itoh et al., 2011; Wrighton et al., 2011). However, neutrophilic Actinobacteria are commonly enriched in metal-reducing conditions (Wang et al., 2009; Lentini et al., 2012) and are detected in 16S rRNA-based surveys of environments where iron reduction is likely occurring (Supplementary Figure S9). One neutrophilic Actinomycetales, *Dietzia* sp. RNV-4, was isolated from the anode of a sediment microbial fuel cell (Sacco et al., 2017). Actinobacteria may therefore be more common contributors to iron reduction than is currently realized.

Although little is known about the Ignavibacteria, there are examples of iron-, arsenic-, and nitrate-reducing species among this group (Podosokorskaya et al., 2013; Kato et al., 2015; Fortney et al., 2016). Other studies have also shown Ignavibacteria spp. to be relatively abundant in anode biofilms (Yoshizawa et al., 2014; Rimboud et al., 2015; Tian et al., 2015; Baek et al., 2016). Our findings of Ignavibacteria genomes rich in large MHCs and porin-cytochrome gene clusters (Figure 3 and Table 3) add further support for EET by members of this group. By searching for porins using four separate HMMs, we could differentiate those homologous to porins from *Geobacter* and *Shewanella* spp., and thereby show the first case of an MtrB-like PCC in Ignavibacteria.

Anaerolineales (Chloroflexi) genomes were rich in MHCs, but over the course of the experiment these organisms were mostly found in the planktonic fraction, making it less clear whether they are able to use the electrode for respiration. However, a Chloroflexi isolate was shown to reduce ferric iron and nitrate (Kawaichi et al., 2013), and is also capable of anodic and cathodic EET (Kawaichi et al., 2018). Other isolates from iron-reducing environments (Hori et al., 2015), and observations that Anaerolineae species make up significant proportions of anode biofilms in MXC studies, suggest this as a possibility (De Schamphelaire et al., 2010; Ishii et al., 2014; Cabezas et al., 2015). Overall, the apparent investment by these organisms in very large MHCs (Figure 5) may indicate that they are capable of some form of EET (Hug et al., 2013).

The genome of S13 Burkholderiales (genus *Hydrogenophaga*) encodes large MHCs as well as PCCs. This, and our identification of a putative PCC from the isolate *Hydrogenophaga* sp. T4, indicate that PCCs may exist in other members of this genus. The large MHCs present in Burkholderiales and Anaerolineales genomes from this study are an intriguing example of the variability in the number of heme-binding motifs in homologous cytochromes (Figures 4, 5). We find very little on this topic in existing literature (Klotz et al., 2008), and highlight it as an exciting unexplored area in multi-domain protein evolution. The rapidly expanding number of (near-) complete genomes will enable a more detailed investigation of this phenomenon.

## Conclusion

Although a novel *Geobacter* species made up the largest fraction of the anode-biofilm consortium enriched from samples of the Rifle, CO aquifer, other bacteria from taxa Actinobacteria, Chloroflexi, Ignavibacteria Betaproteobacteria, and Gammaproteobacteria carried genes for large multiheme cytochromes, putative porin-cytochrome complexes, and/or e-pili in their genomes. A majority of them appear to have the enzymatic pathways needed to utilize acetate as both an electron donor and carbon source. Based on these observations, we infer that these organisms are likely capable of acetate-driven growth using minerals as electron acceptors for respiration, either directly or in association with other species. Our results augment prior metagenomic studies from the Rifle field site, and clarify potential growth strategies for a small subset of organisms present in this environment.

## Data Availability Statement

The datasets generated for this study can be found in the NCBI Sequence Read Archive PRJNA436990.

## Author Contributions

TA, BG, and JB designed the research and wrote the manuscript. TA and BG performed the bioelectrochemical experiments and data analysis. TA and JB processed and analyzed the metagenomic data. All authors contributed to the article and approved the submitted version.

## Conflict of Interest

The authors declare that the research was conducted in the absence of any commercial or financial relationships that could be construed as a potential conflict of interest.

## Footnotes

1 http://dnatech.genomecenter.ucdavis.edu/

2 https://ggkbase-help.berkeley.edu/overview/data-preparation-metagenome/

3 https://sourceforge.net/projects/bbmap/

4 https://github.com/najoshi/sickle

5 http://ggkbase.berkeley.edu/

6 http://eddylab.org/software/ssu-align/

7 http://hmmer.org

8 http://bioinformatics.biol.uoa.gr/PRED-TMBB

9 http://signalfind.org/pilfind.html

10 https://github.com/GlassLabGT/Python-scripts/blob/master/Pilinstats.py
