# Supplementary material for: Diverse Microorganisms in Sediment and Groundwater Are Implicated in Extracellular Redox Processes Based on Genomic Analysis of Bioanode Communities
Source: Front Microbiol. 2020 Jul 28;11:1694. doi: 10.3389/fmicb.2020.01694 (PMC7399161; doi:10.3389/fmicb.2020.01694)
Supplement: Supplementary file 2 [file Data_Sheet_1.DOCX]

**Supplementary Information**


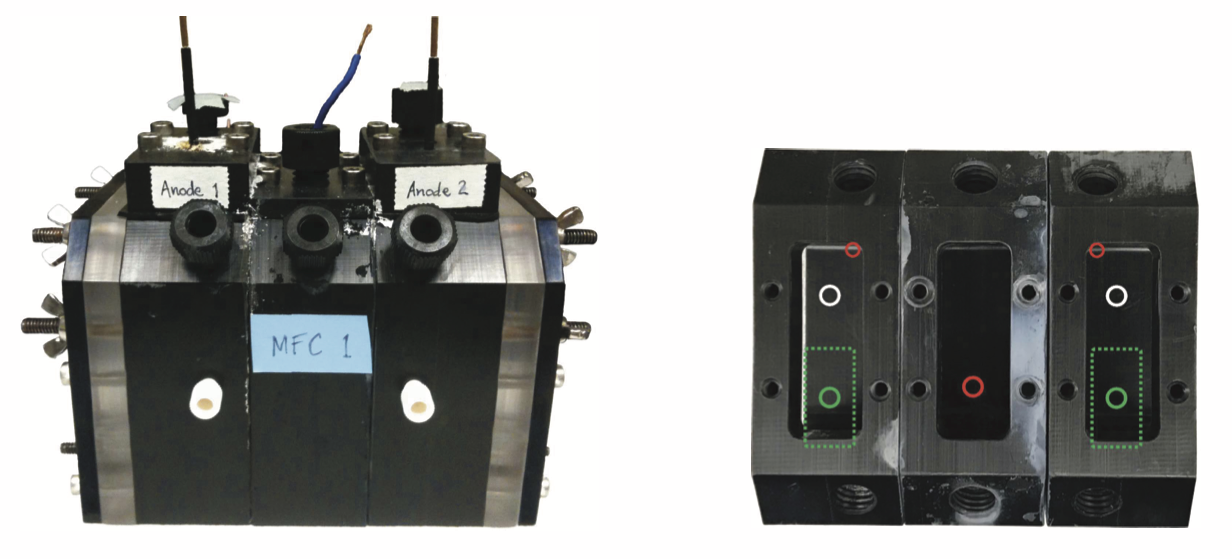


**Figure S1 – Microbial electrochemical cell design.** (**Left**) Front view of the MXC, which has two identical anodic chambers sandwiching a shared cathodic chamber. (**Right**) Top view of the main body of the MXC showing the approximate electrode positions within each compartment: ***green circles and dashed lines*** = graphite anodes (working electrode); ***white circles*** = reference electrodes; ***red circles*** = cathode (central chamber) and additional counter electrodes (outer chambers) used for most cyclic voltammetry analyses.


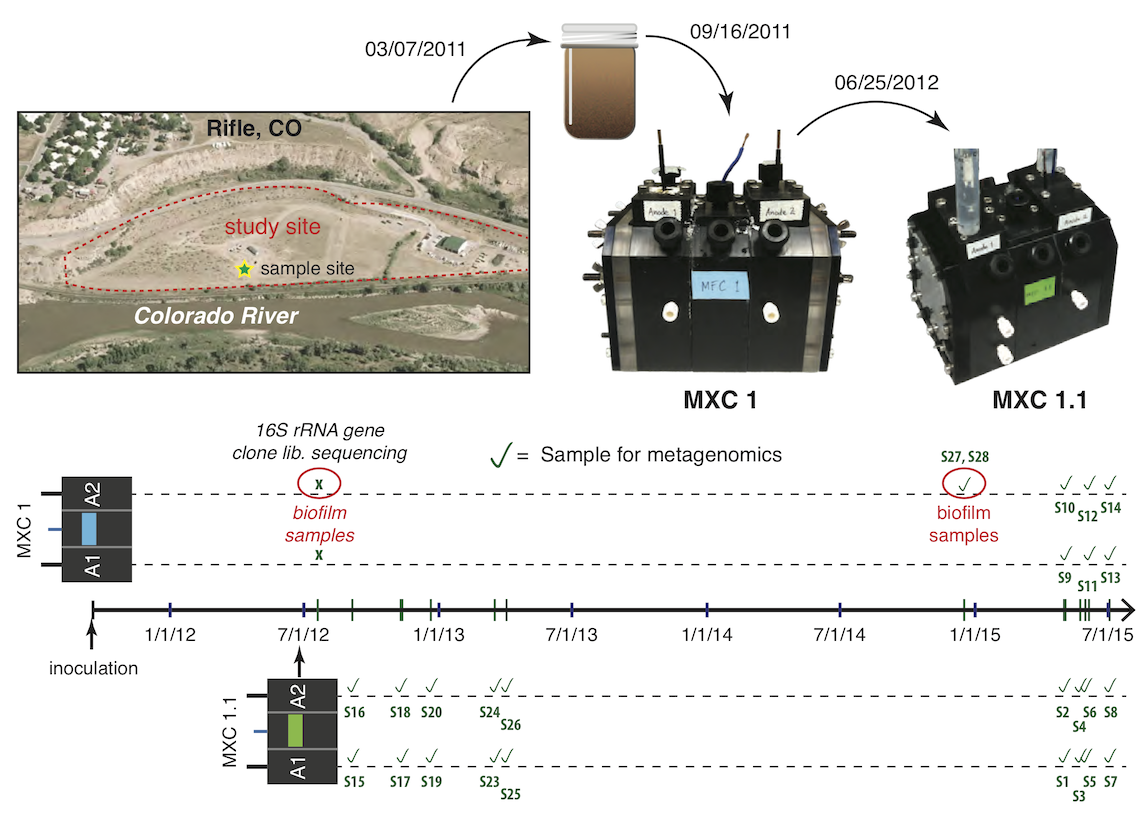


**Figure S2 – Overview of the experiment.** MXC‑1 was inoculated from sediment recovered from the study site adjacent to the Colorado River near the town of Rifle (aerial photograph). Around the time that the biofilm was sampled for 16S rRNA analysis (“X” symbols) a second cell, MXC‑1.1, was inoculated with spent planktonic medium from MXC‑1. Acetate was used as the only electron donor for the entire experiment. Anodic electrodes, which served as the only electron acceptor, were poised mostly at ‑190 mV vs. SHE, a potential within the range of crystalline iron-oxide minerals; the only major exception was a three-month startup period for MXC‑1.1, during which the anodes were poised at a highly favorable potential of +410 mV. The check marks correspond to collection time (also indicated with colored ticks on the timeline) and name of samples, “SX,” from anodic chambers that were analyzed using genome-resolved metagenomics. The key samples in this study are those of the biofilm (S27-28), circled in red.

**A**

**B**

**Figure S3 – First assessments of microbial community composition by 16S rRNA gene clone library sequencing and analysis.** (**A**) Images of anode biofilms collected at 9.5 months of enrichment, showing the sharp color gradation in both. Red-colored regions developed after switching the maintenance potential from +0.4 to ‑0.2 V vs. SHE at around three months, and formed in the same relative location within each anodic chamber. Anodic chambers were not stirred, but the N_2_ gas bubbled into the chambers, which are directly opposed the locations of the red biofilm regions, provided some degree of active mixing. The object seen on the bottom surface of Anode 2 is a rubber gasket accidentally introduced into the chamber. (**B**) Composition of MXC‑1 microbial communities (percent relative abundance). The bar plot is divided into planktonic and anode-biofilm panels because the planktonic sample was collected at four months, and biofilm samples at 10 months. Labels ‘A1’ and ‘A2’ refer to the duplicate anodes; ‘Colorless’ and ‘Red’ refer to the visibly distinct regions of the biofilms that developed on both graphite-block anodes pictured in (A). Taxonomic breakdown is given at the Class or [Phylum] (square brackets) level.

**A**

**B**

**Figure S4 – Electrochemical characteristics of MXC‑1. (A**) Current vs. time for the initial months of the study (sum current from both anodes). Periods of zero current correspond to acetate limitation. The potential poised at the anode was changed from an electropositive startup potential to one more representative of iron reduction (‑190 mV vs. SHE) at around 95 days. This change was followed by a steady increase in current and the growth of visible red-colored biofilms (Figure S1). A test with potential changed back to +200 mV at ca. 118 days (to allow for maximum current production) caused current to increase to the highest value observed in the study. (**B**) Slow-scan-rate cyclic voltammetry collected at 11 months showing the current-voltage relationship for the bioanodes. Two consecutive cycles are shown, and scan rate was 0.02 mV/s (requiring 25 hours for each cycle). The main potential window of current onset between ‑200 and ‑75 mV is consistent with many previous studies of *Geobacter*-dominated anode biofilms. This CV signature remained similar for all such experiments performed over the lifetime of the MXC.

**Figure S5 – Example of current production in MXC‑1.1.** Typical recovery of anodic current following a near-complete exchange of anode and cathode media with the anodic potential poised at ‑230 mV vs. SHE. Poising the anode at a more biologically favorable (electropositive) voltage allowed for significantly higher current. Current is the sum from both anodes (independent tests showed similar magnitude for each). Data collected six months after inoculation.

**Table S1 – DNA sample information and number of sequencing reads generated.** Two independent Illumina HiSeq 3000 runs were performed due to a fluidics problem that occurred during the first run. Fortunately, this issue did not affect the read quality of our samples, and reads from Run 1 and Run 2 were therefore pooled for each sample before assembly and binning.

**Figure S6 – Genome-resolved overview of the composition of MXC‑1 and MXC‑1.1 consortia over time.** Planktonic samples were collected at varying time intervals across a years timespan for the daughter cell, MXC‑1.1. Community composition of those samples is shown in the left side of the figure. Bars appear in groups of two, corresponding to independent samples from duplicate chambers A1 and A2 collected on the same date. Genome order in each bar is according to average abundance in MXC‑1 planktonic samples. The jagged lines separating groups of bars indicate a longer time span or a step back in the timeline. For comparison, the compositional data for the parent cell MXC‑1, shown in **Figure 1** of the main text, is also included (right series of bars, see labels above figure).

**Figure S7 – Maximum-likelihood 16S rRNA gene phylogenetic tree showing the placement of all recovered MXC *Geobacter*/*Pelobacter* sequences including those from preliminary clone libraries.** The tree contains the same sequences as that in **Figure 2** from the main text, with the addition 16S clone-library sequences (shown in red) constructed from anode-biofilm and planktonic samples collected relatively early in the study (see timeline in **Figure S2**). MXC metagenomic sequences appear in bold blue text, and those derived from Rifle samples are colored green. A short descriptor of the isolation source(s) for each organism is included in the sequence name; accession numbers are provided as the last field of each sequence name. Bootstrap values of 50 or greater (based on 100 samplings) are shown in line with their corresponding node.

**Figure S8 – Maximum-likelihood phylogeny of all putative PCC porin proteins from MXC genomes along with closest database hits (NCBI non-redundant database).**

The tree is rooted with a porin protein from a Cyanobacteria. MXC sequences appear in bold blue text. The protein family type is given as the last field in each MXC sequence name, and also indicated by brackets and labels, right side. Bootstrap values (based on 100 samplings) are shown in line with their corresponding node.

**Table S2 – Summary statistics for cytochrome OmcS homologues found in MXC genomes.** Xxxxx… A protein alignment for these 22 sequences is shown in **File S9**.

| Genome | Num sequences | Avg %AA identity | Max %AA identity | Avg Bitscore | Max Bitscore |
| --- | --- | --- | --- | --- | --- |
| S0_RifleGW_Geobacter_53_40 | 3 | 54.1 | 63.5 | 436.3 | 527 |
| S20_RifleGW_Geobacter_56_21 | 4 | 53.9 | 70.0 | 417.8 | 571 |
| S25_RifleGW_Geobacter_56_8 | 6 | 50.1 | 62.0 | 152.4 | 289 |
| S26_Geobacter_56_8 | 4 | 52.2 | 65.2 | 205.8 | 378 |
| S27_BJP_Coriobacteriales_67_81 | 1 | 28.0 | 28.0 | 45.1 | 45.1 |
| S27_RifleGW_Geobacter_56_78 | 4 | 53.7 | 70.0 | 416.3 | 574 |
| *Sum or Avg* | *22* | *51.4* | *NA* | *292.2* | *NA* |

**Table S3 – A comparison of the relatedness of MXC genomes to those reconstructed from the Rifle-site metagenomes using conserved marker genes.** Alignment-based comparisons using all recovered ribosomal protein S3 (RpS3) and 16S rRNA genes from the MXC and Rifle, CO metagenomes were used to evaluate the number of non-redundant (“Unique”) MXC genomes that were phylogenetically similar to those from the Rifle site (35 independently-assembled metagenomic datasets). The analysis was based on USEARCH (Edgar, 2010). The results were filtered at four different identity (ID) cutoffs between ≥ 90% and ≥ 99%. At each %ID cutoff, the numbers for both the “Total” (i.e., redundant) and “Unique” (non-redundant) Rifle genomes are given in separate columns. The bold column headings correspond to the RpS3 and 16S rRNA statistics for the final curated set of MXC genomes. Also shown for 16S rRNA genes are the stats for all recovered/annotated 16S rRNA sequences from MXC genomic scaffolds (includes partially-complete genomes and genes on scaffolds that were not assigned to genome bins), as well as stats for 16S rRNA gene sequences from the preliminary clone libraries (see Supplementary Methods).

**Figure S9 – Maximum-likelihood 16S rRNA gene phylogenetic tree showing the placement of the Coriobacteriales MXC genome within phylum Actinobacteria.** The sequence from the S27 Coriobacteriales genome is shown in bold blue and also indicted with a dot. The tree includes all sequences from the NCBI non-redundant nucleotide database that share ≥ 95% identity, plus a selection of those from cultured Actinobacteria (bold text). Groups of closely related sequences are collapsed into wedges with the number of sequences in each group indicated. A short descriptor of the isolation source(s) for each organism is included in the sequence name, or in italicized text to the right of collapsed clades; accession numbers are provided as the last field of each sequence name. The size of black circles at each node indicates bootstrap value (see legend).

**Figure S10 - Maximum-likelihood rpS3 protein phylogenetic tree showing the placement of the MXC Anaerolineales genomes within phylum Chloroflexi.** Genomes from this study shown in bold blue font. Two of the three (S11 and S13) had identical rpS3 sequences, GC content and genome coverage, and were therefore given one shared label in the tree. Sequences derived from Rifle samples are colored green, and isolate genomes appear in bold text. Groups of closely related sequences are collapsed into wedges with the number of sequences in each group indicated. For uncultured organisms, a short descriptor of the isolation source(s) for each organism is included in the sequence name, or in italicized text to the right of collapsed clades; accession numbers are provided as the last field of each sequence name. The size of black circles at each node indicates bootstrap value (see legend).

**Figure S11 - Maximum-likelihood 16S rRNA gene phylogenetic tree showing the placement of MXC Ignavibacteria sequences.** MXC sequences are shown in bold blue and also indicated with a dot. The two Ignavibacteriales cultured isolates appear in bold, and all other sequences are from uncultured organisms. In addition to the type strains, the tree includes all sequences from the NCBI non-redundant nucleotide database that share ≥ 97% identity with any of the MXC sequences. Sequences from the Rifle, CO site are colored green. Groups of closely related sequences are collapsed into wedges with the number of sequences in each group indicated. A short descriptor of the isolation source(s) for each organism is included in the sequence name, or in italicized text to the right of collapsed clades; accession numbers are provided as the last field of each sequence name. The size of black circles at each node indicates bootstrap value (see legend).

[ See separate file <FigS12_RpS3_all_bacteria.pdf> for figure ]

**Figure S12 – Maximum-likelihood phylogeny of all MXC organisms inferred using ribosomal protein S3.** MXC sequences appear in bold, yellow-highlighted text. The tree also contains closely related sequences from the Rifle-site data (35 independently assembled metagenomes), which are also shown in bold text and are colored by sample type: red = oxygen injection experiments, green = acetate-amended sediment, brown = background sediment, and blue = acetate-amended groundwater. Gray-colored sequences are from the Rifle Carbon Sequestration project (various sample types). Bootstrap values shown at each node are based on 100 samplings.

**Description of Supplementary Files**

**FileS1_ggkbase_listsearchterms_default.pdf**: File containing screen captures showing the current (as of 2020-05-15) search terms used for gene-annotation-based searches on ggKbase (ggkbase.berkeley.edu). These are referred to as “Lists” on the site, and are useful to create a “Genome Summary” for one or many genomes that give a rough metabolic overview. The lists shown here were used to create the metabolic overviews in **File S5** and **S6**.

**FileS2_ggkbase_listsearchterms_tja.docx**: This file shows the equivalent information as File S1, but for the ggKbase “Lists” that were created by the first author (TJA) to perform specific annotation-based searches or to refine the default lists. These lists were iteratively refined by starting with the most general search terms, manually reviewing all results, and adding exclusionary terms and correcting mis-annotations until off-target results were eliminated.

**FileS3_Rubisco_tree.pdf**: Phylogenetic tree showing the placement of genes with “Rubisco” annotations among those from bacterial and archaeal type strains. This was used to classify such genes from MXC genomes (shown in red) into the known Rubisco forms: form I (green) and form II (orange) are most likely to be involved in carbon fixation and were counted in genome summaries (**File S5** and **S6**), whereas forms III (black for bacterial and purple for archaeal types) and IV (grey) have evolved for other purposes and were removed (Tabita et al., 2007). The tree was created in Geneious (Biomatters) using the Jukes-Cantor genetic distance model, neighbor-joining method, and 100 bootstrap re-samplings. Nodes show bootstrap values.

**FileS4_genome_stats.xlsx**: Summary statistics for the final set of curated, de-replicated MXC genomes. Parameters include estimated genome completeness, average abundance in biofilm and planktonic samples from MXC 1 and 1.1, multiheme cytochrome stats including cellular localization predicted by PSORTb, number of putative porin-cytochrome complexes and e-pili. See notes below individual table columns for the meaning of bold or colored text or fill.

**FileS5_metabolism_overview_all.xlsx**: Metabolic overview of all curated non-redundant genomes (n=86) assembled from samples of biofilm and planktonic cell populations in the microbial electrochemical cells. Genome summaries were constructed using ggkbase (<https://ggkbase.berkeley.edu/>) based on annotations of predicted proteins (See *Methods* in main text for details). Columns are grouped into the following metabolic categories: (Complex) carbon degradation, Lipid metabolism, Acetate utilization, Glyoxylate shunt (TCA), TCA cycle, Electron transport chain, Other redox enzymes/complexes, Fermentation, Carbon fixation, H2 metabolism, Methane metabolism, Nitrogen metabolism, Sulfur metabolism. Genomes (rows) were manually grouped according to their “Probable metabolic strategy,” listed in column F. Estimated genome completeness as well as average abundance in biofilm and planktonic samples is also included (columns B-E).

**FileS6_top_EET_genomes_extendedxlsx**: Extended version of Table 1 from the main text, which contains genomes with the greatest number of multiheme cytochromes, potential porin-cytochrome complexes, and putative e-pili. Additional information includes estimated genome completeness, the number of cytochrome OmcS homologues, and a simplified metabolic overview derived from the larger summary of all genomes in **File S5**.

**FileS7_multiheme_cyt_analysis.xlsx**: Input data, results and analysis for predicted subcellular localization of multiheme *c*-type cytochromes (≥ 3 heme-binding motifs) using PSORTb. See also **Table 2**.

**FileS8_putative_ePili.xlsx**: Excel file listing the 86 type-IV pilin protein sequences (N-terminal pre-pilin sequence removed) that we determined, based on the density of aromatic amino acid residues and the lengths of gaps between them, to be considered as putative conductive “e-pili.” See **File S9** for an annotated alignment. See methods and results in main text for more details.

**FileS9_put_epili_align.docx**: Expanded version of **Figure 6** that includes those 20 sequences plus an additional 66 (the same 86 total that are listed in **File S8**) that were confirmed as type-IV by Pilfind (<http://signalfind.org/pilfind.html>), and that also have an anomalously high percentage of aromatic amino acid residues over some length of their sequence. See the main figure and text for further details.

**FileS10_OmcS_homolog_align.pdf**: Protein alignment of sequences found to be homologous to OmcS from *G. sulfurreducens* (WP_119334099.1). The alignment was created in Geneious (v9.1, Biomatters) using MAFFT v7.309 (Katoh and Standley, 2013). Note that the alignment of the 23 sequences spans three rows, and that partial sequences are included that do not appear in every row, indicated by grey text color for the sequence ID at left. Residues are highlighted according to degree of conservation (black highlights indicated most conserved); this is also indicated by the heights (and colors) of the bars and the residue letters in the “Sequence Logo” at the top. Most genomes contain multiple sequences, which are differentiated by the “.N” (where N’s are positive integers) appended to the genome name, in the order that they appear in the alignment. All sequences are from *Geobacter* genomes save the last one from “S27_BJP_Coriobacteriales” (Actinobacteria), which was an abundant member of the MXC‑1 biofilm community with 37 MHCs as well as putative e-pilin genes (**Table 1**).

**References**

Edgar, R. C. (2010). Search and clustering orders of magnitude faster than BLAST. *Bioinformatics* 26, 2460–2461. doi:10.1093/bioinformatics/btq461.

Katoh, K., and Standley, D. M. (2013). MAFFT Multiple Sequence Alignment Software Version 7: Improvements in Performance and Usability. *Mol. Biol. Evol.* 30, 772–780. doi:10.1093/molbev/mst010.

Tabita, F. R., Satagopan, S., Hanson, T. E., Kreel, N. E., and Scott, S. S. (2007). Distinct form I, II, III, and IV Rubisco proteins from the three kingdoms of life provide clues about Rubisco evolution and structure/function relationships. *J. Exp. Bot.* 59, 1515–1524. doi:10.1093/jxb/erm361.
