# Supplementary material for: Diverse Microorganisms in Sediment and Groundwater Are Implicated in Extracellular Redox Processes Based on Genomic Analysis of Bioanode Communities
Source: Front Microbiol. 2020 Jul 28;11:1694. doi: 10.3389/fmicb.2020.01694 (PMC7399161; doi:10.3389/fmicb.2020.01694)
Supplement: Supplementary file 3 [file Data_sheet_2.docx]

**Supplementary Methods**

**Setup of microbial electrochemical cells (MXCs)**

“H-cell” type electrochemical cells with duplicate anodic chambers on either side of a central, shared cathodic chamber (**Figure S1**) consisted of unpolished graphite-block working electrodes (2.5 × 2.5 × 1.0 cm, McMaster-Carr, Chicago, IL, USA) in anodic chambers, a platinum-mesh counter electrode (~1.5 x 1.0 cm) in the cathodic chamber, and additional platinum-wire counter electrodes in anodic chambers used for voltammetry and other three-electrode electrochemical measurements. Reference electrodes were either Ag/AgCl type (3 M KCl; +210 mV vs. SHE for standard conditions, used in MXC-1; Microelectrodes, Inc. model MI-403, Bedford, NH, USA) or saturated calomel (+242 mV vs. SHE for standard conditions, used in MXC-1.1; Accumet, Thermo Fisher Scientific, Waltham, MA, USA). Nafion 117 (DuPont, Wilmington, DE, USA) was used as the proton exchange membrane separating anodic and cathodic chambers.

The first cell, MXC-1, was prepared as follows (Sept 2011): Graphite electrodes were rubbed with a soft sponge in water to remove loose flakes, sonicated once in 5% bleach, then three times in fresh DI water (5 min each time), and stored in DI water until use. Pt electrodes were cleaned by soaking overnight in 1.0-N HCl. Nafion membranes were soaked several times in DI water before cell assembly to dilute the acidity of the hydrated material. The assembled reactor was sterilized by filling with DI water and autoclaving, using a liquid cycle with 20 min at 121 ºC. After cooling, DI water in anodic chambers was replaced with sterile, N2-flushed phosphate-buffered medium, pH 7.2. See main text for medium composition and operation of the MXCs.

A second cell, MXC-1.1, was prepared using the same procedure with the following modifications (June 2012, ~9.5 months after MXC-1 inoculation): Graphite electrodes from above that had been stored in DI water were further cleaned by soaking for 9 hours in 1-N HCl, rinsed and then soaked in DI water for 30 min, soaked in 1-N NaOH for 15 min, rinsed and soaked in acetone for 5 min, then rinsed and stored in DI water until use. For autoclave sterilization, the time at 121 ºC was 15 min. Saturated calomel reference electrodes described above were used in conjunction with homemade salt bridges, which were constructed as described in ref. (Marsili et al., 2008); salt bridges were filled with a 1.5% agar solution containing 0.5-M KCl as supporting electrolyte.

**Nucleic acid isolation and PCR amplification of 16S rRNA genes for phylogenetic analysis (4- and 10-month samples)**

Genomic DNA was isolated from the 4-month planktonic and 10-month anode biofilm samples using a PowerSoil DNA Isolation Kit (MO BIO Laboratories, Inc., Carlsbad, CA, USA) following the manufacturer’s protocol. PCR amplification using universal bacterial primers 27f and 1492r (Weisburg et al., 1991) was conducted with TaKaRa Ex Taq polymerase (Takara Bio, Otsu, Shiga, Japan) with steps as follows: 95 °C for 2 min., 95 °C for 30 s, 52 °C for 30 s, 72 °C for 90 s, 72 °C for 10 min, with steps 2-4 repeated 28 times. Clone libraries were created using the TOPO TA Cloning Kit for Sequencing (Invitrogen/Thermo Fisher Scientific) according to manufacturer protocol and submitted for sequencing at the UC Berkeley DNA Sequencing Facility using M13 vector-specific primers.

**Bioinformatic and phylogenetic analysis of 16S rRNA gene clone libraries**

The Anode 1 red and colorless biofilm samples were sequenced bi-directionally; due to a miscommunication, Anode 1 planktonic and Anode 2 red biofilm samples were sequenced using the forward primer only.

Sequences were quality-filtered and trimmed using Phred, vector-screened with Cross_match, and assembled into contigs with Phrap (Ewing et al., 1998). Contigs less than 300 base pairs were discarded. Contigs were clustered at 99% 16S rRNA gene identity using UCLUST (Edgar, 2010) and checked for chimeras using three methods: 1) UCHIME (Edgar et al., 2011) with the `uchime_ref` option *vs.* both Greengenes and SILVA; 2) `uchime_denovo`, which uses only the sequences in the clone library; 3) DECIPHER (Wright et al., 2012) (<http://decipher.cee.wisc.edu>). If results between different methods were ambiguous, the contig in question was examined manually.

Taxonomy for each sequence was assigned based on the following phylogenetic analysis: Closely related reference sequences were identified using USEARCH (`-ublast` option) (Edgar, 2010) against the GreenGenes (downloaded 9/2013) and Ribosomal Database Project (RDP, downloaded 9/2013; type strains only, good quality) databases. Sequences were aligned using SSU-ALIGN (Nawrocki, 2009) (<http://selab.janelia.org/software/ssu-align>), and a maximum-likelihood phylogeny was inferred using RAxML (Stamatakis, 2006) (<http://sco.h-its.org/exelixis/index.html>) with the GTRGAMMA model of evolution and 100 bootstrap re-samplings.

**References**

Edgar, R. C. (2010). Search and clustering orders of magnitude faster than BLAST. *Bioinformatics* 26, 2460–2461. doi:10.1093/bioinformatics/btq461.

Edgar, R. C., Haas, B. J., Clemente, J. C., Quince, C., and Knight, R. (2011). UCHIME improves sensitivity and speed of chimera detection. *Bioinformatics* 27, 2194–2200. doi:10.1093/bioinformatics/btr381.

Ewing, B., Hillier, L., Wendl, M. C., and Green, P. (1998). Base-Calling of Automated Sequencer Traces Using Phred. I. Accuracy Assessment. *Genome Res.* 8, 175–185. doi:10.1101/gr.8.3.175.

Marsili, E., Rollefson, J. B., Baron, D. B., Hozalski, R. M., and Bond, D. R. (2008). Microbial biofilm voltammetry: direct electrochemical characterization of catalytic electrode-attached biofilms. *Appl. Environ. Microbiol.* 74, 7329–37. doi:10.1128/AEM.00177-08.

Nawrocki, E. P. (2009). Structural RNA Homology Search and Alignment Using Covariance Models.

Stamatakis, A. (2006). RAxML-VI-HPC: maximum likelihood-based phylogenetic analyses with thousands of taxa and mixed models. *Bioinformatics* 22, 2688–2690. doi:10.1093/bioinformatics/btl446.

Weisburg, W. G., Barns, S. M., Pelletie, D. a, and Lane, D. J. (1991). 16S ribosomal DNA amplification for phylogenetic study. *J. Bacteriol.* 173, 697–703.

Wright, E. S., Yilmaz, L. S., and Noguera, D. R. (2012). DECIPHER, a Search-Based Approach to Chimera Identification for 16S rRNA Sequences. *Appl. Environ. Microbiol.* 78, 717–725. doi:10.1128/AEM.06516-11.
