# Supplementary material for: Diverse Microorganisms in Sediment and Groundwater Are Implicated in Extracellular Redox Processes Based on Genomic Analysis of Bioanode Communities
Source: Front Microbiol. 2020 Jul 28;11:1694. doi: 10.3389/fmicb.2020.01694 (PMC7399161; doi:10.3389/fmicb.2020.01694)
Supplement: Supplementary file 4 [file Data_Sheet_3.zip › FileS1_ggkbase_listsearchterms_default.pdf]

Acetate Production *alternate*

Created by: Universal

conversion of acetyl CoA to acetate, can also do butyrate with 92% efficiency in pyrococcus

Match **ANY** of these terms

- × rci:RCIX831 acdAB; acetyl-CoA synthetase
- × EC 6.2.1.3
- × acyl-coa synthetase
- × acetyl-CoA synthetase
- × acetyl coenzyme A synthetase (ADP formin
- × ADP-forming
- × Acetate--CoA ligase

Match **ALL** of these terms

Exclude these terms

- × acid adenylation protein

Acetate Production (ACK-PTA)

Created by: Universal

Match **ANY** of these terms

- × acetate kinase
- × phosphotransacetylase
- × EC 2.7.2.1
- × butyrate kinase
- × ackA
- × K00925
- × phosphate acetyltransferase
- × EC 2.3.1.8

Match **ALL** of these terms

Exclude these terms

Alcohol_DH_Ethanol_Production

Created by: Universal

Match **ANY** of these terms

- EC:1.1.1.1
- alcohol dehydrogenase

Match **ALL** of these terms

Exclude these terms

- aldehyde-alcohol dehydrogenase
- acetaldehyde dehydrogenase
- ankyrin repeat-containing protein
- EC:1.1.1.14
- molecular chaperone GroES
- PQQ repeat protein

Aldehyde_DH_Ethanol_Production

Created by: Universal

Match **ANY** of these terms

× aldehyde dehydrogenase

Match **ALL** of these terms

Exclude these terms

AMP_salvage_pathway_RuBisCO

Created by: Universal

Match **ANY** of these terms

Match **ALL** of these terms

× ribulose

× bisphosphate

× carboxylase

Exclude these terms

ATP synthase_Complex_V

Created by: Universal

Match **ANY** of these terms

× ATP synthase × ATP synthetase

Match **ALL** of these terms

Exclude these terms

× dethiobiotin

Beta_oxidation_Fatty_acids_Acetyl-CoA_acetyltransferase

Created by: Universal

Match **ANY** of these terms

× Acetyl-CoA acetyltransferase

Match **ALL** of these terms

Exclude these terms

Beta_oxidation_Fatty_acids_Acyl-CoA_Dehydrogenase

Created by: Universal

Match **ANY** of these terms

× Acyl-CoA dehydrogenase

Match **ALL** of these terms

Exclude these terms

Beta_oxidation_Fatty_acids_Acyl-CoA_synthetase

Created by: Universal

Match **ANY** of these terms	Match **ALL** of these terms	**Exclude** these terms
× acyl-coa synthetase		

Beta_oxidation_Fatty_acids_enoyl_hydratase

Created by: Universal

Match **ANY** of these terms	Match **ALL** of these terms	**Exclude** these terms
× Enoyl-CoA hydratase		

Blue_copper_protein

Created by: Universal

Match **ANY** of these terms

Match **ALL** of these terms

× blue

Exclude these terms

Cellulose Degradation_3.2.1.21_3.2.1.4_3.2.1.91

Created by: Universal

beta glucosidase (3.2.1.21)

Match **ANY** of these terms

× ec 3.2.1.21 × 3.2.1.4 × 3.2.1.91

Match **ALL** of these terms

Exclude these terms

Complex_III

Created by: Universal

Match **ANY** of these terms

× 1.10.2.2

× quinol-cytochrome c reductase

× K00412 × K00413 × K00411

Match **ALL** of these terms

Exclude these terms

Complex_II_Succinate_Dehydrogenase

Created by: Universal

Match **ANY** of these terms

× succinate dehydrogenase
× 1.3.5.1

Match **ALL** of these terms

Exclude these terms

× tricarballylate dehydrogenase

Complex_I_NADH_Dehydrogenase

Created by: Universal

Match **ANY** of these terms	Match **ALL** of these terms	**Exclude** these terms
× NADH ubiquinone oxidoreductase		× NAD-dependent epimerase
× nadh dehydrogenase		× NAD-dependent epimerase/dehy
× 1.6.5.3		
× NADH-quinone oxidoreductase		

Cytochrome_c

Created by: Universal

Match **ANY** of these terms

Match **ALL** of these terms

× cytochrome c

Exclude these terms

× quinol × soxd × oxidase

× biogenesis × reductase

× peroxidase × assembly

× 1.1.2.8 × quinone

Cytochrome_c_oxidase_subunit II_Complex_IV

Created by: Universal

Match **ANY** of these terms	Match **ALL** of these terms	**Exclude** these terms
× coxB<br>× cytochrome c oxidase subunit II<br>× cytochrome c oxidase subunit 2		

Cytochrome_c_oxidase_subunit_I_Complex_IV

Created by: Universal

Match **ANY** of these terms	Match **ALL** of these terms	**Exclude** these terms
× cytochrome c oxidase subunit I<br>× coxA		

Dissimilatory_Sulfate_Reduction

Created by: Universal

Match **ANY** of these terms

- sulfate adenyltransferase
- 1.8.99.2
- dsrB
- dsrK
- dissimilatory sulfite
- 2.7.7.4
- dsrA
- adenylylsulfate reductase
- sulfite reductase

Match **ALL** of these terms

Exclude these terms

- elongation factor

DMSO_Molybdopterin_oxidoreductase

Created by: Universal

Match **ANY** of these terms	Match **ALL** of these terms	**Exclude** these terms
× Molybdopterin oxidoreductase		

electron_transfer_flavoprotein

Created by: Universal

Match **ANY** of these terms

× electron transfer flavoprotein

Match **ALL** of these terms

Exclude these terms

Formate_Dehydrogenase

Created by: Universal

Match **ANY** of these terms

× formate dehydrogenase

Match **ALL** of these terms

Exclude these terms

Glycolysis

Created by: Universal

Match **ANY** of these terms

- × glyceraldehyde 3-phosphate
- × 2,3-bisphosphoglycerate-independent phos[illegible]
- × 2,3-bisphosphoglycerate
- × bisphosphoglycerate
- × Glucose 1-dehydrogenase
- × glucokinase
- × phosphoglucose
- × Phosphofructokinase
- × fructose bisphosphate aldolase
- × fructokinase
- × fructose-bisphosphate aldolase
- × enolase
- × phosphoglucomutase
- × pyruvate kinase
- × pyruvate phosphate dikinase
- × hexokinase
- × glucose-6-phosphate isomerase
- × triosephosphate isomerase
- × Glyceraldehyde 3-phosphate dehydrogena[illegible]
- × phosphoglycerate kinase
- × phosphoglycerate mutase
- × deoxyribose phosphate aldolase

Match **ALL** of these terms

Exclude these terms

- × N-acetyl-gamma-glutamyl-phosp[illegible]
- × Alpha-ribazole phosphatase
- × Deoxyribose-phosphate aldolase
- × heterodixulfide reductase subuni[illegible]
- × polyferredoxin

Glycosyl_hydrolase

Created by: Universal

Match **ANY** of these terms

- cellobiosidase
- glycosidase
- glycosyl hydrolase
- endoglucanase
- glycoside hydrolase
- cellulase
- chitinase
- 2.4.1.18
- glycogen debranching enzyme
- galacturonase
- mannosidase
- arabinase
- glucuronidase
- xyloglucanase
- xyloglycosyltransferase
- mannanase
- xylanase
- xylosidase
- arabinofuranosidase
- galactanase
- galactosidase
- glucoronidase
- rhamnosidase
- fucosidase

Match **ALL** of these terms

Exclude these terms

Heterodisulfide_reductase_ABCD

Created by: Universal

Match **ANY** of these terms	Match **ALL** of these terms	**Exclude** these terms
× heterodisulfide		

Lactate_Dehydrogenase

Created by: Universal

Match **ANY** of these terms

- × Lactate/malate dehydrogenase
- × lactate dehydrogenase
- × D-isomer specific 2-hydroxyacid dehydroge[illegible]

Match **ALL** of these terms

Exclude these terms

Methane_oxidation

Created by: Universal

Match **ANY** of these terms

- × 1.14.18.3
- × particulate methane monooxygenase
- × methane monooxygenase
- × 1.14.13.25

Match **ALL** of these terms

Exclude these terms

Methanogenesis_McrA

Created by: Universal

Match **ANY** of these terms	Match **ALL** of these terms	**Exclude** these terms
× 2.8.4.1		× ribosomal protein
× methyl-coenzyme M reductase alpha subur		
× methyl coenzyme M		
× Methyl-coenzyme M reductase		

NitrogenFix_NifHDK

Created by: Universal

Match **ANY** of these terms

× nifH × nifD × nifk × EC:1.18.6.1 × nitrogenase

Match **ALL** of these terms

Exclude these terms

× sam × trna × elongator × elongation × chromosome partitioning × NifS × septum × mind × ATPase

Nitrogen_Cycle_ammonia_monooxygenase

Created by: Universal

Match **ANY** of these terms	Match **ALL** of these terms	**Exclude** these terms
× 1.14.99.39<br>× ammonia monooxygenase		

Nitrogen_Cycle_hydroxylamine_oxidoreductase

Created by: Universal

Match **ANY** of these terms

× hydroxylamine oxidoreductase

× 1.7.3.4

Match **ALL** of these terms

Exclude these terms

Nitrogen_cycle_Nitrite_reductase

Created by: Universal

Match **ANY** of these terms

Match **ALL** of these terms

× nitrite reductase

Exclude these terms

Nitrogen_cycle_Putative_Nitrate_reductase

Created by: Universal

Match **ANY** of these terms

Match **ALL** of these terms

× nitrate reductase

Exclude these terms

Pyruvate_dehydrogenase

Created by: Universal

Match **ANY** of these terms

× pyruvate dehydrogenase

Match **ALL** of these terms

Exclude these terms

Quinone_reductase_complex

Created by: Universal

Match **ANY** of these terms	Match **ALL** of these terms	**Exclude** these terms
× Polysulphide reductase<br>× Molybdopterin oxidoreductase		

RNF_complex

Created by: Universal

Match **ANY** of these terms	Match **ALL** of these terms	**Exclude** these terms
× rnfc × rnfa × rnfb × rnfd × rnfe × Rnfg		

Sulfite_reductase

Created by: Universal

Match **ANY** of these terms

× sulfite reductase

Match **ALL** of these terms

Exclude these terms

Sulfur_oxidation_sox_pathway

Created by: Universal

Match **ANY** of these terms

× sulfur oxidation × sulphur oxidation
× soxA × soxb × soxc × soxd
× soxz × soxy × soxx × soxW

Match **ALL** of these terms

Exclude these terms
