# Supplementary material for: Diverse Microorganisms in Sediment and Groundwater Are Implicated in Extracellular Redox Processes Based on Genomic Analysis of Bioanode Communities
Source: Front Microbiol. 2020 Jul 28;11:1694. doi: 10.3389/fmicb.2020.01694 (PMC7399161; doi:10.3389/fmicb.2020.01694)
Supplement: Supplementary file 4 [file Data_Sheet_3.zip › FileS2_ggkbase_listsearchterms_tja.docx]

**Tyler Arbour’s custom lists on ggKbase**

**(2020-05-17, 12:30 PM GMT)**

[**Acetate kinase**](https://ggkbase.berkeley.edu/lists/48745-Acetate-kinase)

**ANY:** acetate kinase, 2.7.2.1

**Created by:** [you](https://ggkbase.berkeley.edu/lists/user/28) on 2020-05-05

[edit](https://ggkbase.berkeley.edu/lists/48745-Acetate-kinase/edit)

[**Acetate transporters**](https://ggkbase.berkeley.edu/lists/48743-Acetate-transporters)

**ANY:** acetate permease, cation/acetate symporter, actp, sodium solute transporter, sodium/solute symporter
**NOT:** sodium/glucose

**Created by:** [you](https://ggkbase.berkeley.edu/lists/user/28) on 2020-05-04

[edit](https://ggkbase.berkeley.edu/lists/48743-Acetate-transporters/edit)

[**Acetyl-CoA transferase**](https://ggkbase.berkeley.edu/lists/48747-Acetyl-CoA-transferase)

**ANY:** 2.3.1.9, acetyl-CoA C-acetyltransferase, acetoacetyl-CoA thiolase, beta-acetoacetyl coenzyme A thiolase, 3-oxothiolase, acetyl coenzyme A thiolase, Acetyl-CoA acetyltransferase, acetyl-CoA:N-acetyltransferase, thiolase II, type II thiolase
**NOT:** 2.3.1.16

**Created by:** [you](https://ggkbase.berkeley.edu/lists/user/28) on 2020-05-06

[edit](https://ggkbase.berkeley.edu/lists/48747-Acetyl-CoA-transferase/edit)

[**Fe-Hydrogenase_tja**](https://ggkbase.berkeley.edu/lists/48770-Fe-Hydrogenase_tja)

**ANY:** hydrogenase, NAD(P)-dependent iron-only, Fe-hydrogenase, [Fe] hydrogenase
**NOT:** nickel-dependent, maturation protein, nifE, maturation, expression, nickel, small, PAS, hyfB, hydrogenase 4, 3, methyl-viologen, Molybdenum, Ni,Fe-hydrogenase, assembly, Glutamate synthase (NADPH), NADH:ubiquinone oxidoreductase, nadh dehydrogenase, NADH:ubiquinone reductase, transcriptional, expression/formation, Ni/Fi, Ni/Fe hydrogenase, thiamin-monophosphate kinase, Thiamine-monophosphate kinase, dsrO, accessory, hypothetical protein, formate hydrogenlyase, uncharacterized protein, NADP oxidoreductase, N-methylhydantoinase A, hydantoinase, HypD, NADH-ubiquinone oxidoreductase, nrfD, polysulfide reductase, Polysulphide reductase, arxB, hypC, HypE, methyl-viologen-reducing

**Created by:** [you](https://ggkbase.berkeley.edu/lists/user/28) on 2020-05-14

**Copied from:** [Fe-Hydrogenase](https://ggkbase.berkeley.edu/lists/5340-Fe-Hydrogenase)

[edit](https://ggkbase.berkeley.edu/lists/48770-Fe-Hydrogenase_tja/edit)

[**Heterodisulfide_reductase_ABCD COPY**](https://ggkbase.berkeley.edu/lists/48772-Heterodisulfide_reductase_ABCD-COPY)

**ANY:** heterodisulfide

**Created by:** [you](https://ggkbase.berkeley.edu/lists/user/28) on 2020-05-15

**Copied from:** [Heterodisulfide_reductase_ABCD](https://ggkbase.berkeley.edu/lists/5473-Heterodisulfide_reductase_ABCD)

[edit](https://ggkbase.berkeley.edu/lists/48772-Heterodisulfide_reductase_ABCD-COPY/edit)

[**Hydrogenase_NiFe_tja**](https://ggkbase.berkeley.edu/lists/48771-Hydrogenase_NiFe_tja)

**ALL:** hydrogenase
**ANY:** nickel-iron, nickel-dependent, nickel, [NiFe], (NiFe), Ni/Fe
**NOT:** expression/formation, HypD, insertion, isoenzyme, assembly, maturation, expression, hypA, hypB, hypC, HypE, hypF, HupF_HypC, accessory, formation, HupV, HupU, transporter, nickel-transporter, HoxM

**Created by:** [you](https://ggkbase.berkeley.edu/lists/user/28) on 2020-05-14

**Copied from:** [Hydrogenase_NiFe](https://ggkbase.berkeley.edu/lists/5455-Hydrogenase_NiFe)

[edit](https://ggkbase.berkeley.edu/lists/48771-Hydrogenase_NiFe_tja/edit)

[**Isocitrate lyase**](https://ggkbase.berkeley.edu/lists/48761-Isocitrate-lyase)

**Description:** Enzyme 1 of 2 for glyoxylate shunt / bypass in TCA cycle for carbon fixation from acetate / acety...

**ANY:** isocitrate lyase, isocitrase, isocitritase, isocitratase, threo-Ds-isocitrate glyoxylate-lyase, isocitrate glyoxylate-lyase, 4.1.3.1
**NOT:** methylisocitrate, isocitrate lyase family protein, transposase

**Created by:** [you](https://ggkbase.berkeley.edu/lists/user/28) on 2020-05-11

[edit](https://ggkbase.berkeley.edu/lists/48761-Isocitrate-lyase/edit)

[**Malate synthase**](https://ggkbase.berkeley.edu/lists/48762-Malate-synthase)

**Description:** Enzyme 2 of 2 for glyoxylate shunt / bypass in TCA cycle for carbon fixation from acetate / acety...

**ANY:** malate synthase, 2.3.3.9, L-malate glyoxylate-lyase, glyoxylate transacetylase, glyoxylate transacetase, glyoxylic transacetase, malate condensing enzyme, malate synthetase, malic synthetase, acetyl-CoA:glyoxylate C-acetyltransferase

**Created by:** [you](https://ggkbase.berkeley.edu/lists/user/28) on 2020-05-11

[edit](https://ggkbase.berkeley.edu/lists/48762-Malate-synthase/edit)

[**Multiheme Cytochromes**](https://ggkbase.berkeley.edu/lists/10470-Multiheme-Cytochromes)

**ALL:** cytochrome
**ANY:** multi-haem, multiheme, multi-heme, multihaem
**NOT:** napC, nirN, sulfite oxidase, class I, biogenesis, assembly, Cytochrome bd oxidase, K02275, succinate dehydrogenase, Menaquinol, nitrite, cox, coxC, oxidase, coxA, coxB, coxD, ubiquinol, lactate, p450, cytochrome b, cytochrome b5, Cytochrome b6, cytochrome B561, dehydrogeanse, dehydrogenase, cytochrome c553, cytochrome d1, molybdopterin, class ii, hydrogenase, trimethylamine-N-oxide reductase, methylamine, cytochrome c peroxidase, thiosulfate reductase, napb, nitric oxide, nirb, hydroxybutyrate, nirm, cytochrome c-551, cytochrome c551/c552, cytochrome c5, cytochrome c2, cytochrome c556, nirC, sulfite, cycM, cytochrome c6, cycI, c-556, monoheme, pedF, c-550, c550, diheme, b562, p 450, cytochrome c1, K02034, LysR, nrfa, soxx

**Created by:** [you](https://ggkbase.berkeley.edu/lists/user/28) on 2016-02-03

[edit](https://ggkbase.berkeley.edu/lists/10470-Multiheme-Cytochromes/edit)

[**PFOR**](https://ggkbase.berkeley.edu/lists/48750-PFOR)

**Description:** Search terms include all KEGG synonyms for EC 1.2.7.1. Exclusions were added by manually going th...

**ANY:** 1.2.7.1, Pyruvate oxidoreductase, pyruvate synthase, Pyruvate synthetase, pyruvate:ferredoxin oxidoreductase, Pyruvic-ferredoxin oxidoreductase, 2-oxobutyrate synthase, Alpha-ketobutyrate-ferredoxin oxidoreductase, 2-ketobutyrate synthase, Alpha-ketobutyrate synthase, 2-oxobutyrate-ferredoxin oxidoreductase, 2-oxobutanoate:ferredoxin 2-oxidoreductase
**NOT:** Indolepyruvate, 1.2.7.8, 2-ketoisovalerate, 1.2.7.7, acetoin, EC:1.1.1-, Pyruvate decarboxylase, 4.1.1.1, Ketoisovalerate, 2-oxoacid:acceptor oxidoreductase, 1.2.7.3, uncharacterized, phosphoenolpyruvate, 2.7.9.2, pyruvate carboxylase, putative oxidoreductase, pyruvate phosphate dikinase, Pyruvate, phosphate dikinase, 2-isopropylmalate synthase, pyruvate/ketoisovalerate, putative pyruvate, water kinase, 2-oxoglutarate:acceptor oxidoreductase, carbamoyl-phosphate synthetase, FAD-dependent oxidoreductase, isopropylmalate

**Created by:** [you](https://ggkbase.berkeley.edu/lists/user/28) on 2020-05-06

[edit](https://ggkbase.berkeley.edu/lists/48750-PFOR/edit)

[**Phosphotransacetylase**](https://ggkbase.berkeley.edu/lists/48748-Phosphotransacetylase)

**Description:** Also known as "phosphate acetyltransferase."

**ANY:** 2.3.1.8, phosphate acetyltransferase, phosphotransacetylase
**NOT:** 1.1.1.40, malate dehydrogenase

**Created by:** [you](https://ggkbase.berkeley.edu/lists/user/28) on 2020-05-06

[edit](https://ggkbase.berkeley.edu/lists/48748-Phosphotransacetylase/edit)

[**Porin-cytochrome porins**](https://ggkbase.berkeley.edu/lists/12046-Porin-cytochrome-porins)

**Description:** High similarity to Geobacter OmbB (GSU2733) based on HMM search, and near MHCs ==> likely part of...

**ALL:** porin
**ANY:** OmbB, ExtB, ExtI, mtrB

**Created by:** [you](https://ggkbase.berkeley.edu/lists/user/28) on 2017-03-24

[edit](https://ggkbase.berkeley.edu/lists/12046-Porin-cytochrome-porins/edit)

[**Pyruvate carboxylase**](https://ggkbase.berkeley.edu/lists/48760-Pyruvate-carboxylase)

**ANY:** pyruvate carboxylase, pyruvic carboxylase, 6.4.1.1
**NOT:** oxaloacetate, biotin carboxylase, methylcrotonyl-CoA carboxylase, carbamoyl-phosphate synthetase, acetyl-CoA carboxylase, accC, 6.3.4.14

**Created by:** [you](https://ggkbase.berkeley.edu/lists/user/28) on 2020-05-11

[edit](https://ggkbase.berkeley.edu/lists/48760-Pyruvate-carboxylase/edit)

[**Pyruvate dehydrogenase**](https://ggkbase.berkeley.edu/lists/48744-Pyruvate-dehydrogenase)

**Description:** Copy of AJP's list.

**ANY:** Pyruvate decarboxylase, Pyruvate:lipoamide 2-oxidoreductase, Pyruvic acid dehydrogenase, Pyruvic dehydrogenase, Pyruvate oxidase, pyruvate dehydrogenase, [EC:1.2.5.1], [EC:1.2.4.1], [EC: 1.2.5.1], [EC: 1.2.4.1]

**Created by:** [you](https://ggkbase.berkeley.edu/lists/user/28) on 2020-05-05

**Copied from:** [Pyruvate dehydrogenase](https://ggkbase.berkeley.edu/lists/8204-Pyruvate-dehydrogenase)

[edit](https://ggkbase.berkeley.edu/lists/48744-Pyruvate-dehydrogenase/edit)

[**RNF_complex COPY**](https://ggkbase.berkeley.edu/lists/48773-RNF_complex-COPY)

**ANY:** rnfc, rnfa, rnfb, rnfd, rnfe, Rnfg

**Created by:** [you](https://ggkbase.berkeley.edu/lists/user/28) on 2020-05-17

**Copied from:** [RNF_complex](https://ggkbase.berkeley.edu/lists/5472-RNF_complex)

[edit](https://ggkbase.berkeley.edu/lists/48773-RNF_complex-COPY/edit)

[**Wood-Ljungdahl_ACS**](https://ggkbase.berkeley.edu/lists/48766-Wood-Ljungdahl_ACS)

**ANY:** 2.3.1.169, acetyl-CoA synthase, acetyl coA synthase
**NOT:** carbamoyl-phosphate synthase

**Created by:** [you](https://ggkbase.berkeley.edu/lists/user/28) on 2020-05-12

[edit](https://ggkbase.berkeley.edu/lists/48766-Wood-Ljungdahl_ACS/edit)

[**Wood-Ljungdahl_COD**](https://ggkbase.berkeley.edu/lists/48765-Wood-Ljungdahl_COD)

**ANY:** carbon monoxide dehydrogenase, 1.2.99.2, carbon-monoxide dehydrogenase, carbon-monoxide dehydrogenase (ferredoxin)
**NOT:** aerobic, aerobic-type

**Created by:** [you](https://ggkbase.berkeley.edu/lists/user/28) on 2020-05-12

[edit](https://ggkbase.berkeley.edu/lists/48765-Wood-Ljungdahl_COD/edit)

[**Wood-Ljungdahl_FDH**](https://ggkbase.berkeley.edu/lists/48764-Wood-Ljungdahl_FDH)

**ANY:** 1.17.1.9, formate dehydrogenase

**Created by:** [you](https://ggkbase.berkeley.edu/lists/user/28) on 2020-05-12

[edit](https://ggkbase.berkeley.edu/lists/48764-Wood-Ljungdahl_FDH/edit)

[**Wood-Ljungdahl_MTHFD**](https://ggkbase.berkeley.edu/lists/48767-Wood-Ljungdahl_MTHFD)

**ANY:** 1.5.1.5, methylenetetrahydrofolate dehydrogenase, 1.5.1.5 3.5.4.9, 5,10-methylene-tetrahydrofolate, 6.3.4.3, 3.5.4.9, methenyltetrahydrofolate cyclohydrolase, Citrovorum factor cyclodehydrase, formyl-methenyl-methylenetetrahydrofolate synthetase, 5,10-methenyltetrahydrofolate 5-hydrolase, formate--tetrahydrofolate ligase, Formyltetrahydrofolate synthetase, 10-Formyl-H4 folate synthetase, 10-formyltetrahydrofolate synthetase

**Created by:** [you](https://ggkbase.berkeley.edu/lists/user/28) on 2020-05-12

[edit](https://ggkbase.berkeley.edu/lists/48767-Wood-Ljungdahl_MTHFD/edit)

[**Wood-Ljungdahl_MTHFR**](https://ggkbase.berkeley.edu/lists/48768-Wood-Ljungdahl_MTHFR)

**ANY:** 1.5.1.20, methylenetetrahydrofolate reductase, 5,10-methylenetetrahydrofolate reductase, 5,10-methylenetetrahydrofolic acid reductase, 5,10-CH2-H4folate reductase, 5-methyltetrahydrofolate:NAD+ oxidoreductase

**Created by:** [you](https://ggkbase.berkeley.edu/lists/user/28) on 2020-05-12

[edit](https://ggkbase.berkeley.edu/lists/48768-Wood-Ljungdahl_MTHFR/edit)

### Search Help

The search box searches a snapshot of the information in the database and does not support wild cards (e.g. '*' or '%').

You can search by gene name as follows:

- full gene name : UBA_LeptoII_Scaffold_8241_GENE_539
- contig/scaffold abbreviated : UBA_LeptoII_Scaff_8241_GENE_539 (or "cont" for contig)
- locus_id : UBAL2_82410539
- just scaffold or contig and a number : 8241_539
- organism and scaffold/contig and number : LeptoII_8241_539

You can also search by annotation - you can enter any word that might be found in the annotation fields. If the annotation for a gene is Ferredoxin oxidoreductase gamma subunit 517295:517993 forward MW:25534 you would get hits for any word:

- Ferredoxin
- oxidoreductase
- gamma
- subunit
- 517295:517993
- forward
- MW:25534

Any combinations of the above words work as well

- oxidoreductase gamma subunit

Finally, you can add in the organism name as well. To only find those oxidoreductases from A-plasma, you might search for either of these:

- aplasma oxidoreductase
- apl oxidoreductase
