# Supplementary material for: Diverse Microorganisms in Sediment and Groundwater Are Implicated in Extracellular Redox Processes Based on Genomic Analysis of Bioanode Communities
Source: Front Microbiol. 2020 Jul 28;11:1694. doi: 10.3389/fmicb.2020.01694 (PMC7399161; doi:10.3389/fmicb.2020.01694)
Supplement: Supplementary file 4 [file Data_Sheet_3.zip › FileS9_put_epili_align.docx]

Genome Mature pilin sequence

Residue number: 1 10 20 30 40 50 60 70 80 90 100 110 120 130 140 150

(single digits) 123456789**X**123456789**X**123456789**X**123456789**X**123456789**X**123456789**X**123456789**X**123456789**X**123456789**X**123456789**X**123456789**X**123456789**X**123456789**X**123456789**X**123456789**X**

Key AA positions: * * * # $$ #

*G. metallireducens* PilA (WP_004511668.1, 15.3%, 22) FTLIELLIVVAIIGILAAIAIPQFAAYRQKAFNSAAESDLKNTKTNLESYYSEHQFYPN

S20_RifleGW_Geobacter_56_21 (11.3%, 22) FTLIELLIVVAIIGILAAIAIPQFSNYRVKSYNSAAVSDLKNSKTALESFYADNQRYPGLTD

S25_RifleGW_Geobacter_56_8 (15%, 22) FTLIELLIVVAIIGILAAIAIPQFSAYREKAYNSAAQSDGKNAKTAFEAFFADNQKYPFK

S27_RifleGW_Geobacter_56_78 (15%, 22) FTLIELLIVVAIIGILAAIAIPQFSAYREKAYNSAAQSDGKNAKTAFEAFFADNQKYPFK

S28_RifleBG_Geobacter_53_18 (13.6%, 22) FTLIELLIVVAIIGILAAIAIPQFSAYREKAYNSAAQSDGKNAKTALEAFFADNQKYPF

S28_RifleBG_Geobacter_53_18_seq2 (13.6%, 22) FTLIELLIVVAIIGILAAIAIPQFSAYRAKAYNSTSQSDLKNNKTALESFFADNQQYPY

S0_RifleGW_Geobacter_53_40 (11.5%, 22) FTLIELLIVVAIIGILAAVAIPQFSSYRIKGFNSNATSDVRNFRTTLEAVYADRQGYPGGN

S28_RifleBG_Geobacter_53_18_seq3 (10.4%, 30) FTLIELMIVLSIIGILSSIAVPNYQRWIIRAKETALSDSLNNFRKTIDEYYADQGKYPDELNELVARGYLRGLPIDPFTKKSDTWVTVAPPEGNTLPLDSGSQPVTAPVKEPGNVYDVHSGSDLVGSNGVPYNEW

S12_RifCSP_Betaproteobacterium_64_16 (10.7%, 22) FTLIELMIVVVVIGILSAVAIPAYTDYVTRGKLVEGTSMLSDGRVKMEQFFQDNRTYVGGPAPAATTYFTYAASNLTADTYTITATGIGSLAGFVYTIDQTNTKRTTSAPAGWAAAAMPAACWISKKGGGC

S16_RifleBG_Stenotrophomonas_66_10 (10.6%, 35) FTLIELMIVVIVIGILAAIAIPSYQQYVLRSHRAVAKADLAEYAQRAERYHSSNNSYSGYTLPSKVSPREGGATRYDLSYKGDGSTFTITASPSGTQVKDSCGKLTLDQANRKTSGGAVSDCW

S1_Delftia_acidovorans_67_20 (10.5%, 22) FTLIELMIVVAVIVILGTVAVPSYNEYIRGVHRADARAGLLQAQQWLERAATANGVYPTTLPVDLTWARDSSKRYTIGFLATSGHDDDTYTLIATRRGSQLNDRCGDFTLTHTGERSGANVASSTTAAECWGR

S1_Delftia_acidovorans_67_20_seq2 (9.8%, 22) FTLIEMMIVVAVVGILTAIAIPSYSEYIRRGHRADARAGLLQAQQWLERASTATGVYPTALPTTLTWAGDNSKRYTIGFAANNSNASYTLTAVPRGPQTGDKCGTYTLSNTGLRGADGKVSSDSAFKADCWGK

S15_RifCSP_Burkholderiales_59_53 (9.6%, 22) FTLIEVMIVVAIIGILTAIAIPSYSEYIRRGHRADARAGLLQAQQWLERASTATGSYPTALPSTRTWAGDATKRYTIAIGGPATTSSFTLTATPRAGTPQAGDKCGNYTLTHTGVRGANGKIQSDAGFDATCWNK

S21_RifCSP_Burkholderiales_65_185 (9.6%, 22) FTLIEVMIVVAIVGILTAIAIPSYSEYIRRGHRADARAGLLQAQQWLERASTATGNYPTTLPASMTWTADNTKRYAIAIGGPATTTSFTLTATPKAGTPQVGDKCGTYTLTHTGIRGANGLIQTDASFDATCWNK

S7_RifCSP_Burkholderiales_64_14 (9.6%, 22) FTLIEIMIVVAITGILAAVAIPSYAEYIRRGHRADARAGLLQAQQWLERASTATGTYPTALPANLTWAADPTKRYTIAIGGPATTSSFTLTATPKAGTPQAGDKCGNYTLTHTGIRGANGKIQTDAGFDATCWNK

S23_RifCSP_Comamonadaceae_63_35 (9.2%, 22) FTLIEVMIVVAIVGILTAIAVPSYNEYIRRGHRAEGRAGLLQAAQWMERAATATGTYPLAAAFPATLKTIPSDRYDITVASADGRTFTLTATPKGAHTGDKCGNYTLTQAGVRDASPLTMGATVAECWGK

S9_RifleAAC_Dechloromonas_62_369 (12.4%, 19) FTLIEVMIVVAIIGILSSIAYPAYDEHVRNARRTAAMGCLMEQTHFMERFYSANMTYVGAALPACPGQVTTFYNLGTANLGATTYTLTAAPTGAQAGDRCGTFSVTHTGTRTNSTAINCWQ

S4_RifCSP_Pseudomonas_61_25_seq3 (9.5%, 22) FTLIELLTAVAIVAVLAGVVYPAYVGHVNKAYRAEIVMLLTEQAQSLERYYTSNGSFIDASGVSVGNDRYRITAALNPQDFSLLATPGVDSVMAGDACGAFSLTSSGARTNPGAAPGMSRKTCWGQ

S15_Burkholderiales_66_31 (12.3%, 34) FTLIEMLIALAVIGILSALAYPSYRDHVLRARRSEAFNALSTVAQAQDRWRATHTRYADSLADLGLESISNPGRYYQIELQTHVPHSHSQFTAIARATGTQRADVRCTLLQLGQNGPQQLRTASDHLGQDTSRHCWPS

S1_Delftia_acidovorans_67_20_seq3 (9.2%, 22) FTLIELMITVAVVAILSAIAYPSYQEYVLRSRRVEAQSLLGEAAARQERWRAQNGGYTSSVANLRLPWGDKSENGHYTLTMATTANDGGYTLKATRTGRQASDRRCGDYTLNALGVKGISPLGTVEDCWR

S7_Holophaga_63_20_seq2 (12.4%, 20) FSLLELLVAMTILAVIGTIGFVQFRTHTARARHIKARSNVEIIGQGLDHYYLKHGFYPMLGSFEAMVEANSPLVKENFIPVNSPSKDPWDQPLEGSSTKVSYVLKCAGDPGNPEEFGPFTIEPGKYSDSSQPSAPAAGATPEAPK

Genome Mature pilin sequence

Residue number: 1 10 20 30 40 50 60 70 80 90 100 110 120 130 140 150 160 170 180

(single digits) 123456789**X**123456789**X**123456789**X**123456789**X**123456789**X**123456789**X**123456789**X**123456789**X**123456789**X**123456789**X**123456789**X**123456789**X**123456789**X**123456789**X**123456789**X**123456789**X**123456789**X**123456789**X**

Key AA positions: * * * # $$ #

*G. metallireducens* PilA (WP_004511668.1, 15.3%, 22) FTLIELLIVVAIIGILAAIAIPQFAAYRQKAFNSAAESDLKNTKTNLESYYSEHQFYPN

S15_RifleAAC_Geothrix_68_9 (10.6%, 22) FTLMELLVVMTIMALLATVGLAGYRYKVQVAREAVLKENLFQINHALEQYRADRGKYPSSLGPLRELGYLRDIPWDPITQSRDTWQLELEPPDPDNPEGETGVFRVRSGSTDKGTNQIPYNEW

S15_RifCSP_Pseudomonas_62_25_seq3 (8.7%, 25) FTLIEIMIVIAIIGLVLTLSLPSITEYMKKPHRSEIAVLLTEQAQQLERHFSKTGAYSNAPDLSTGNEYYSITPTLSDSGFSLTATPKADGMMAGDKCGSFSIDQTGATSISGQADGLAAKDCWGR

S20_RifleGW_Geobacter_56_21_seq2 (13.4%, 22) FTLIELMIVMTIVGILAAIAVPNYKWGVIKAKEAVLKEELYNWGTVIDHFYADHGKYPDSLQELVEKKYLRDIPKDPITKSKETWVVLPPPSPEDGGEVKGSVYEVHSGSDLVGSDGRPYNEWRYGD

S26_Geobacter_56_8 (13.4%, 22) FTLIELMIVMTIVGILAAIAVPNYKWGVIKAKEAVLKEELYNWGTVIDHFYADHGKYPDSLQELVEKKYLRDIPKDPITKSKETWVVLPPPSPEDGGEVKGSVYEVHSGSDLVGSDGRPYNEWRYGD

S27_RifleGW_Geobacter_56_78_seq2 (13.4%, 22) FTLIELMIVMTIVGILAAIAVPNYKWGVIKAKEAVLKEELYNWGTVIDHFYADHGKYPDSLQELVEKKYLRDIPKDPITKSKETWVVLPPPSSEDGGEIKGSVYEVHSGSDLVGSDGRPYNEWRYGD

S0_RifleGW_Geobacter_53_40_seq2 (9%, 39) FTLIELMIVVSIIGILAAVAAPNYNRGIIKAREAVLSEDLYNFRTTIDQFYADQGKYPDTLNELTEKGYMRGLPKDPFTKQSDTWVTIAPPAESSTTSSPLLGSTDQMGGGQSVMGGGSGPGNVYDVKSGSNLVGTNGVPYNEW

S0_RifleGW_Geobacter_53_40_seq4 (9.9%, 49) FTLIELLIVIALIGILLSAATFGFSQYSRKSKMESQTRFLYGDLMEYRTKAMYEKRNWTFKMSAAGYGIYSSTNTDVSPVTAVVLKHPVDVASTVTEIEFDTHGLTDNIGSVCVTSANNAVVDSVVISKTRVQIGKKREGTNCVSTNIDAK

S26_Geobacter_56_8_seq3 (9.7%, 22) FSLVELLTVVATMIILLSVATLQFSSMMRKRDMEKEIRMIYTDLSEVRQKALYEKTPRAVKFTANEFTVYPGASTTASPVLRRSLNHPVGWSTADSVLVVTFDT

S0_RifleGW_Geobacter_53_40_seq5 (9.4%, 26) FTLIELIVVIFLIATLMAVAGLNFSNWRTKYKVEAQVKEMAADINELRLMAMTSKKKHSITLNANSYVFRSYSSDDEPITSGTILTGGTHNVSYGLKSGATTTFNNNRLEIDQQGLLVSSDDTIIYLDRSDVAFLNCLTLQTARTNVGSRNATWSNCDDR

S28_RifleBG_Geobacter_53_18_seq6 (8.4%, 42) FSLVELVTTIGIISILLAIATLNYNNWQVKYNVENQVKVMHSDINSLRLRAIHTKKLHRIELQSGGYVFKRSSSDEDVYVSGPIVESKTVTYPLMKADGTSINGENIDFNSRGFTSNNLTIRIDSPTNNAAQDCLIVSAAQTNLGKMENGTCTPK

S13_RifleGW_Desulfovibrio_67_8 (8.7%, 24) FTLIELIAVIIILGILSAVIVPKYFDMTGKATEGAANAAISEGVARFNMGYASYILNTSAKPANLAALAPNYVNATMDLGDWAVGLSSDGTTVTIGAYDNKAGTIAEAETGVPSGTATVSKSFPWPQ

S14_RifleGW_Desulfovibrio_65_8 (9.8%, 22) FTLIELISVIIILGILAAVITPKYFDMTSKAQDAAYKGALSEGVARLNMGYAAYILGESKKP

S13_RifleGW_Desulfovibrio_67_8_seq2 (9.4%, 24) FTLIELIAVVVLLGILAAIVVPRYIGFVEESRKAVASAALSEGLSRFRMGYVKFVLATADRPGDFAALGDSYVNATINLGDFAAGLSQDGDTMTIGAYDNKARTISDAVAGVPSGAPVVSKAFPWPR

S7_Holophaga_63_20 (10.4%, 22) FTLLELVTAMTILALLTSVGIVGYRHQTRTAREAVLKEDLFQLNHALECYRADRGKYPASIGRLRELGYLRDIPVDPMTGSRDTWTEENEAQDPDNPDAEVGIFRIRSGSTDVGSNGIPYNEWSY

S22_Xanthobacter_67_227 (10.6%, 25) YTLVELLVVITIIGLIVALVGPRVLGYLGDSKVKTAKIQIQGFSSALDLFYLDAGRYPSSSEGLNALVQRPGNVSTWNGPYLKGGTVPLDPWGKAYAYRAPGQHGPFDIVSLGSDGQEGGTGNAADVTSWAR

S22_Xanthobacter_67_227_seq2 (10.6%, 25) YTLVELLVVITIIGLIVALVGPRVLGYLGDSKVKTAKIQIQGFSSALDLFYLDTGRYPSTSEGLAALVQRTPGAATWNGPYLKGGTLPSDPWGKPYSYRAPGEHGAYDIVSLGSDGQQGGTGTAADITSWSK

S9_Rhizobiales_66_65 (8.9%, 41) FTLVEMLVVLAIIGLISALVGPRVLSQLSDSRERAARLQIEAFSSALDIFFIDVGRYPVEAEGLGALMQKPASIQTWNGPYLRGESVPLDPWGREYRYSSDGRSFRIVTQGADGRASGAAQIR

S4_RifCSP_Pseudomonas_61_25 (9.9%, 48) FTLIEIMVVVVILGILAAMVVPKVLDRPDQARATAAKQDIGGLMQALKLYRLDHGTYPTMNQGLKVLVERPADAKNSNWRAYLDRLPNDPWGHPYHYLNPGANGEVDVFSLGADGQPDGDGVNADIGSWQL

S15_RifCSP_Pseudomonas_62_25 (9.2%, 48) FTLIEIMVVVVILGILAAMVVPKVLDRPDQARATAAKQDIAGLMQALKLYRLDHGSYPNMNQGLKVLVERPANAKDSNWRSYLERLPNDPWGRPYHYLNPGANGEVDVFSLGADGQPDGDGVNADIGSWQL

S15_RifCSP_Pseudomonas_62_25_seq2 (9.6%, 48) FTLIEIMVVVVILGVLAALVVPQIMSRPDQAKGAAAQSDIKAIAMALDIYKLDNHQYPSSQQGLEALVSKPSGNPPARNWNPDGYLKRLPIDPWGNAYQYRVPGARGTGYDLFSFGADGKLGGDGLNAEIGNWDR

S21_RifCSP_Pseudomonas_stutzeri_64_159 (9.7%, 48) FTLIEIMVVVVILGILAALVVPQVMSRPDQAKVTVAKGDIKAIAAALDMYKLDNFAYPSTQQGLDALVKKPSGNPQPKNWNRDGYLKRLPKDPWGNDYQYLSPGTQGQFDLYSFGADGKPGGSDLNADIGNWDL

S20_RifleGW_Geobacter_56_21_seq3 (8.8%, 48) FTLIEIMVVIVILAALAALVGPKIMGRTDDAKIADAKVQIRNIETALKLYKLDSGNYPSTEQGLTALVTKPATGIIPRNYKPEGYLESKQVPKDPWGAEYIYLAPGEHGEYDLYSLGADGTRGGEGKNADIESWNLR

S27_RifleGW_Geobacter_56_78_seq3 (8.8%, 48) FTLIEIMVVIVILAALAALVGPKIMGRTDDAKIADAKVQIRNIETALKLYKLDSGNYPSTEQGLTALVTKPATGIIPRNYKPEGYLESKQVPKDPWGAEYIYLAPGEHGEYDLYSLGADGTRGGEGKNADIESWNLR

S28_RifleBG_Geobacter_53_18_seq4 (9.5%, 48) FTLIEIMVVIVILSMLAILVGPKIIGRTDDAKIADAKIQIKNLETALKLYKLDNGDYPGTEQGLQALVEKPTAGVIPKNYREGGYLEHKDVPKDPWDNDYLYLSPGEHGDFDLYSLGADGVRGGESKDADIESWNLR

S12_Novosphingobium_64_29 (8.7%, 38) FSLVELMVVIFIIGLLATVVLINVLPSQDKAMSVKARSDIATLEQAMDMYRLDMATYPNQAEGIAALKSPPAGLALPQNYRSGGYVKDVPLDPWGRPYQYQVPGRDGKPFEIFSLGADGQPGGTDQNADIYSGQN

S9_Caulobacterales_69_30 (9.6%, 48) FTLVELMVVIVIIGLLATVVVINVLPSQDRAMIGKARADISVLEQAIETYRLDNLTFPDDQQGLQALVAPPAGLARPDRYRQGGYVRRLPEDPWGNPYQYRRQSAHGGQFDVYSWGADGREGGEGDDADLGNWQA

S4_RifCSP_Pseudomonas_61_25_seq2 (12.6%, 31) FTLLEMLAVIVLLGIVATIVVRQVGGNVDKGKYGAGKAQLASLSMKIDSYALDVGSPPNSLQQLVDKPGNASSWAGPYAKLSELKDPFGHAFGYRFPGEHGAFDLIFYGQDGQPGGDGYSADLGNWE

S27_BJP_Coriobacteriales_67_81 (10.9%, 22) FTLVELMVVVLIIGILVAIAIPVFNAASRTARQRTCHSNQRTIEGAIQQWLAASPTNVWTPKLINGADELTTNGAYIKDVPQCPIATATQFYATNTSGTITFDGSTAWLVDGTLTHGHF

S27_BJP_Coriobacteriales_67_81_seq2 (9.6%, 25) FTLVELMVVVLIIGILVAIAIPVFVAASRTAAERTCQTNQRTIEGAVQQWIAGDPARWWSAQVIDGATDALTDPTAPYLLAPPRCPNAEALFYGVDASGTVTADDLAAGDPVPGVWTSATGHDHY

S12_Firmicutes_42_19 (10.7%, 35) FTLIELIIVMAIISILSYIILPSITSSLQIGHNSADESNLQVLNSTTQFLRTTMKGNDPFEDPENSSEDLMNVLINKKLLPSKLKPLTAGKEFAWFREQGKWDYSDATLGDSDEDDDDDGGVIVDPPGDEEPPVSGPQPWKSGTGYKLGDEVIYNGTLYQARKDTTSTPGTINGDWQEI…

…TSEYRNFNLYATGDEVVFDGKVFVARGTTFNEIPGQVSSPWQEITNQWRNFNVYQKGDEVIYNGTTYRAKQNRNAGDDTNPTNPKFWQALK

S0_RifleGW_Geobacter_53_40_seq3 (9%, 27) LSLVELVVTVAILTILASAVIPLSHMTARRTREIELRRNLRTIRTAIDDYKKKYEWALENKKINEVVNKSSGCPENLQILVEGYDFGELTPPKRKFLRRIPPDPMNPPGPGEEPKWGLRSYNDKPDSTSWGGEDVFDIYSLSEGTAIDGTKYKDW

S28_RifleBG_Geobacter_53_18_seq5 (9.7%, 49) VTLLELIVAISILTILVAGIVPLTRMTAKRTREIELRSNLRIIRTAIDDFKKSYDKAVDDKKIPVTENKSGYPETLEQLVEGYDFGGLYAFKKKFLRKVPVDPMNPPEPGEEPEWGMRSYSDQPDSSSWGGEDVYDVYSLSEGTALDGTKYGEW

S20_RifleGW_Geobacter_56_21_seq4 (8.5%, 49) VSLIELVVTVAILSVLASLILPSAQLITKRTKELELRRNLRTMRTAIDEYYKAYQDAVYVSKTMPIPSAADPKASGYPESLQVLVEGFDFGKVDGAKKRFLRRIPIDPFNPPAPGEEPKWGLKGYADDPDDDPKETPEEIDGGLFDVVSLSEETAIDGTKYNEW

S26_Geobacter_56_8_seq2 (8.5%, 49) VSLIELVVTVAILSVLASLILPSAQLITKRTKELELRRNLRTMRTAIDEYYKAYQDAVYVSKTMPIPSAADPKASGYPESLQVLVEGFDFGKVDGAKKRFLRRIPIDPFNPPAPGEEPKWGLKGYADDPDDDPKETPEEIDGGLFDVVSLSEETAIDGTKYNEW

S27_RifleGW_Geobacter_56_78_seq4 (8.5%, 49) VSLIELVVTVAILSVLASLILPSAQLITKRNKELELRRNLRTMRTAIDDYYRAYQDAVYVSKTMPIPSAADPKASGYPESLQVLVEGFDFGKVDGAKKRFLRRIPVDPFNPPAPGEEPKWGLKGYADDPDDDPKETPEEIDGGLFDVVSLSEETAIDGTKYKEW

S13_RifOxy_Acholeplasmatales_34_21 (9.9%, 39) VTLVELLAVVVILGIIAAIAVPTIGGLIARQQANADTATYNAIVDAAELYGGTAVFTLDKLETDDFIDLKTNTFSFDGETPVAKTAVYIKITGGVVGFYSDLAGTVAVDFYVNDTLVYEKP

S23_Clostridiales_37_15 (13.5%, 45) FTLIEMIVIIVILAIISIIAIPTVLGLNAEAEKQVCETNRDTILRMYQIYCAQETDCSLVDFFDLDVDKIITSAICPSHGDFTPTGQGITANITCSIHDGGGEPSPDPDPDPVFPLIPGTDVYVNSTWPADEEFVGSNGYNQVVWVHGGQTYFYQGQYYVVSGNVDLFSNTGNPYPTPI…

…NGWWYNNNSVGVIKISDKRWNFTGSTTEEFQAMTGTSSGSVNKGDVCTWQGKTYVFTNATYGWVAPPHNNSDWVEIKTQL

S24_RifOxy_Desulfosporosinus_43_10 (9%, 28) FTLIEIMLVIAVIGVLAVVTVPKYQAVTNQSHLESSAQKVVGQLRYAKQLAMDQRETIYLVMDTNTVRVLDARNKEYGGSQAFDNGVNFDKTSAESNGLTVKSDMMSGLPYVEYDYHGFVIEETHVPGVSIKVVLSGKYNTITIVVEPQTGNIKXXXXXXXXXXXD

S12_RifleGW_Bacteroidetes_47_42 (12%, 40) FSMSELLVVLVIIGILVLIALPNLMPLISKAKSVEAQQQLVFLHSLQQSYFFTHSKYSLSLDELGFEQQPLVSEDGTANYKIEIVEADENGYRAQAMAVVDFDRDGTFNTWEIDQNKHLRETERD

S5_RifOxy_Bacteroidetes_44_7 (12.6%, 40) FSMSELLVVLVIIGILVLIALPNLMPLISKAKSVEAQQQLVFLHSLQQSYFFTHSKYSLSLDELGFEQQPLVSEDGTANYKIEIVEADENGYRAQAMAVVDFDRDGTFNTWEIDQNKHLRETERDICNSFYL

S1_Delftia_acidovorans_67_20_seq4 (8.7%, 37) FTAIELMVVVAILAVLTTLAAPSFHLIIERWRVRQTVEGLQSTLQYARSEAVRRGGGVFIQKLPQGTNGCTLAARHADWGCGWVVFVDRNGNRRWDPREELQRFDTPARTLVIRSRSAAIISVDRWGQMGGFTAMGFAVAPSSAGLVSAAAKGVCVSSGGRMRVVGREGIPCA

S7_RifCSP_Burkholderiales_64_14_seq2 (9.1%, 34) FTVIELMVVVSILALLAALALPSFTPLIERWRVQQAVKGLESTLYYARAEAIKRGGTVTVRKHPTGTGGCLLAPGNANWDCGWFVFTDTNSNGTLDAGEEVLQSFPTPPNIDVTRVGGAASIEFDRWGRVSGPFVGFSLVPHDKNISDLAARGLCMSSGGRIRTIKSEDMPCTSG

Residue number: 1 10 20 30 40 50 60 70 80 90 100 110 120 130 140 150 160 170 180

(single digits) 123456789**X**123456789**X**123456789**X**123456789**X**123456789**X**123456789**X**123456789**X**123456789**X**123456789**X**123456789**X**123456789**X**123456789**X**123456789**X**123456789**X**123456789**X**123456789**X**123456789**X**123456789**X**

Key AA positions: * * * # $$ #

*G. metallireducens* PilA (WP_004511668.1, 15.3%, 22) FTLIELLIVVAIIGILAAIAIPQFAAYRQKAFNSAAESDLKNTKTNLESYYSEHQFYPN

S5_RifleAc_Desulfovibrio_putealis_63_18 (10.5%, 29) FTLIELLVTILLVSVLSAMTLPLFFSGVTRSSDPLNQMPTPLSLQDIMARIIADYYSNATSYLHDLNLLNANITTGNYGITAGHTITKDPAYKFDPSDIGTALKVTIRDNTTGQTMTYVFTKQL

S24_RifOxy_Desulfosporosinus_43_10_seq2 (13.7%, 17) FTLWELMLVFFLMGVIMTLVTPHFGSATNQVWIRVDLANRARIEGATQLYRIDVGTYPQSVSDLVHVPNEVSRWRGPYLDNIPINPFDSAQIYQIDALGQIK

S14_RifleGW_Desulfovibrio_65_8_seq2 (8.9%, 42) FTLIEVITVLLIIGILSAVILTRGGSLNSDLPARMGEVRSQLRYLQLMAMKNGISYLVLTCDGTDYWAYNSADANKHLPLPGESAAKIPLADKKMQMSPFTISFDAFGIPYNGSPQVKLAHNATISITVGGQTDSLSVTPETGFVP

S2_Clostridiales_33_24 (10.9%, 21) FTLIEVVATVAIASLVMVALVSFLSVVSKVFVASAIESEQRLIASSAKAYLKNELTYVTEIAVNGDGSFSKLEFTGGRIYKNDVQVFDNEFYGNTSIYGQVTGLGSVLTFTIRVENGGTNRTEDYVIKTLNKVDTAISTPVTTLYYK

S15_RifCSP_Pseudomonas_62_25_seq4 (10%, 29) FTLLEVMVALAIFATVSIALFSAIQHVAINSGNLAERTQATWIADNYLNELRSGMQPRTLGRQQRQVEFGGRNWWLFSEIEAAPDSRLLKVNLRVSAEQDPQRARQYSRAQLLGYIEANP

S12_RifCSP_Betaproteobacterium_64_16_seq2 (9.2%, 40) FTLVEVLVALAIISIALLSALRAAGQGTNNVGELRSRLLAGWVAENLLAEHRARADWLPLGIQRGSSRQGGVDFAWREEVIATPNPAFRRVDVRVYATAEESHSLSHLAGFIVNAPGVRR

S0_RifleGW_Geobacter_53_40_seq6 (8.6%, 35) FTLLEVLIAVVLLGILSSALYGSYFAVLRARDRASEGMESRRELGGTLDLLRREIASMPLYNRNDKRLRFVVEDRDNFGKPASNLEMTTLAPPSTQVRGESGIINVQYRMLEKNKRFLLMRREHDAQLELTTVPSYPQMEQINAFLVECYDGSKWVKSWDTSLNGRLPKIVRITVQIEE…

…NGKPVEFSVYSDPKVTGS

S1_Delftia_acidovorans_67_20_seq5 (11.4%, 36) FTLLELLVAFALMSLLVLGLAGAMSTVSSTSQRVESRLDLADQQRVHSGFLRAVLGNISAVKRQGGTFKPNQSQFVFSGAGDSIEWLGYLPPGAGAAGRQHMRLDVGPLADGSAGLVLHFMPWQGPEVDPNWGQAQTYVLERDVQGFVLSYRDAGRGDWLPAWTEPRYLPAAISVRVAT…

…RQGGWPLLAVAVHSPLLTNPGAESEIGFGSRY

S15_RifCSP_Pseudomonas_62_25_seq5 (8.8%, 25) FTLLEVVVAIAIFSLLGLATYQLLDRVMRSDQRIQTQEQQLRHLQRALSLLERDLVQVQRHALKDDHSHSQALISQHQGLRLLRGGWRNPLDAPRSDLLQVNHRLSDGAWIRETQGLERDSLSQVQPLLSGVELIHLRFIDALGQPHDSWPIGSEALSLPAAVDIELSAPGFPHIRRVI…

…LLPGGAEVDEDAPRE

S26_Geobacter_56_8_seq4 (9.1%, 29) LTLVELLIVTLILGLISTALMIVFISGQYEYRVRDATVRMQQQARLAMTILERELKMTGYGLMDLGSLKINRYMGGSAPLKIAVIEAQDGGNSGGPDAITVSFQNPNKDTDLHQNIVVTKDYPSSKPDTLFVSSIDRFSAGDLFLIFDPTDLTKPASMLQVSHMPGSDKMLKHTSNSYN…

…PPNNFELFPQIATDNTYTTN

S12_Firmicutes_42_19_seq2 (9.8%, 20) FTLIELIITMFLLTVVLGLVTGIVTNSLKFFSDERTQVNNQASLRLIAVDFEKDVRKYVLGIDQFSYTSGCYRIVPELVSDINYCLVGTNLTRNGVLIGEQVSQFTAVYNATNNSIALTIRSLPDAYGRVNEVIVNIYIRILS

S1_Delftia_acidovorans_67_20_seq6 (10.4%, 45) FTLIETLVATVVTALGVLGILSLQMRTLADTQSGVRRAQAIRLIEDFSERTHANPNSLGQMGNYESDWDHTPESTADCSATPCDPETLASYNLALWKASVHQLLPSGAAKVFPATDDTSNGRHLGIVISWRENEKSSKAGYKKPIGLHSDSTHGDEAMACPTGRTCHLQYIALSARCAP…

…YLANNAVQFFCASP

S7_RifCSP_Burkholderiales_64_14_seq3 (9.1%, 35) ITLIESLVAIVVAALGILGVLGVQMRTLSDTQTSVHRAQAIRLIEDLSERMKTHPNALVSIDSYTVGWRPGPAPTPQASKLCEGATNCTHAEFAAYDLREWKRTLERTLPLGDASVFFAPAETTAGSRRQLGVMIRWRENERSTDNSYLADLLTTATNLGGDAGMPTCNVSPTSSPRYT…

…CHLQYIPVSSRCAPYFADVTVQYFCPGPKS

S20_RifleGW_Geobacter_56_21_seq5 (10.9%, 22) FTLVELLVAILLLMIGFLAVITVFWTSAASGTFTRQMTTAASLGEEMLERAKTLSYNSLGTTGGFVNYTAANASARSFTRRWSITESGGVKTITAEISWGGGTMGTKTRTFTMTKRSDY

S25_RifleGW_Geobacter_56_8_seq2 (10.9%, 22) FTLVELLVAILLLMIGFLAVITVFWTSAASGTFTRQMTTAASLGEEMLERAKTLSYNSLGTTGGFVNYTAANASARSFTRRWSITESGGVKTITAEISWGGGTMGTKTRTFTMTKRSDY

S26_Geobacter_56_8_seq5 (10.9%, 22) FTLVELLVAMLLLMIGFLAVITVFWTSAASGTFTRQMTTAASLGEEMLERAKTLSYNSLGTTGGFVNYTAANASARSFTRRWSITESGGVKTITAEISWGGGTMGTKTRTFTMTKRSDY

S27_BJP_Coriobacteriales_67_81_seq3 (11.4%, 26) FTLSELIVVVGLLGMVLGIAYAGFSVAASGSRMSDRQAYLSREVGAPLEFADRVLTQAFDFDTSYPGLNPNRFAFYTDQDSDGNRERYVIEVVGTRLLVTSEEEGGGRPRRQVVWSEHNANLAAGEPLFRYYDMHGTQITSMGDLPGNAKRIVMTIVTEHDGTRLKDGREMFLRNR

S4_Rhizobiales_62_21 (12.5%, 37) FSLVELSIVLVILGLLTGGILAGQSLIRAAELRSVTTEYGRWVTAMHSFRDKYMGIPGDMRDATRFWGHQTTTGWCSNTSGAAVSVNGTCDGNGNGTISLTAAANQGGENFAFWRQLALAGLIEGTYSGVAGPTNAEDSIIAVNIPSSKFSQAGWSSWGGDFPGDPYSFRYNYGIGFTY…

…GAAGGQKAQGRIMKPEEAWNIDMKMDDGRPATGKVVARFWNNECSIPNSGAASNTNYDASYNLGDSAIRCALHFPNLF

S4_Rhizobiales_62_21_seq2 (11.6%, 40) FSLVELSIVLVILGLLTGGILAGQSLIRAAELRTVTTDLQKYQTAIRSFQDKYMALPGDMTNATRFWGVRAVGTNIACQQTINSYDGTCNSDGNGQIDYIAGDISLGERFLAWQHLALAGLIEGSYTGASGSTTSGAHLRGVNTPPSRLGDAFFSLSHITSPQSGHANWFDGNYGFNTL…

…WLAGVSGRALRPDELWSIDNKLDDGKPATGYVFTVKSTGSYAPGCTTTDVASTSEYAVTSNSKLCTGYFVLR

S23_Azospira_oryzae_66_856 (8.7%, 40) FTLIEAIVAMVLLGIAGSLVGMFIRIPIEGYFDTERRARLTDTADTALRRMARDLRLALPNSIRITAVGNVRYLEFLQTRVGGRYRTDPTAAGGGNPLEFGIADTNGFDVLGTPPVFQAGDHVAIANLGTDSGADAYTGNNMVPSTGITPAGVVQFAAFRFPVPSPGARFFIVDRRVTY…

…ECNPDVGVLRRYSGYALVGPAQPTPPGVAPVLLAQRVANRANACTMTYDANANTRMGIVSMSLTLEEAGESVTLLHQVHVSNVP

S9_RifleAAC_Dechloromonas_62_369_seq2 (8.8%, 34) FSLLELSVVLIVFGLLAGSMLGNLSGQRQLAEEQRARRQLELSLEALYGFAISHGRLPCPAEPALNSDLADAGNESCPLEHGVLPWRSLGLAETDPWGQRLSYYARREFTMSPAADARAGFMLDSEGNANIRPAASAGNKLADKLPAVIVSHGRNGSSGYRSNGQPTPAGHPDESENAD…

…ADLIYVNRLPDDHYDDLVTWIIPAILNARMLAAGRLP

S15_Burkholderiales_66_31_seq2 (11%, 26) VTLIEALITVFLLCLLAFIALPTWQSHLLQKRLESAVETYRQHFQWARSHAMRSGQTVSIRFASDATGSCYIVFTGPEAACGCNAAAAQCAAPARLLVSEHLPADRHIVMQPKSVDKTFSLGPLHGTVTPTPVIVFSAPNGQTLHEVANRLGRTRSCSPQGSLPGWPACTTT

S13_RifOxy_Acholeplasmatales_34_21_seq2 (10.5%, 21) LTLVELLGAVVIFGISISLIALLLSVIFNANDKILEQSRANTEGTIVIAHLEDLMRNFAVTDYSTCIDNPNCVTLESHYTYELSGDSSSIILVKHTPPNTLQISILNNQLYINGVVHEIRNFEIHSDSYIEKTTVNNQLKLKIVIILYINEGKTYTFTSNQTFDLSDVPAS

S24_RifOxy_Desulfosporosinus_43_10_seq3 (12.4%, 18) FTLLEVMITISIFGLLMLYASQFMRSEVNVFQSVSRQNEVEQKARIAMMHIVDEVRLTNLTFYKSTSNNQGIYRYANAAAAALRDETDSTSLIFIKLPSSSDSPPSSAKVFFDYDNSEGEGTLWYIKNGFQYLIADEISQLSIIPDATDEHLVKIDIXXXGKNRSRPYELLTWVRLY
