# Supplementary figures and images for: Diverse Microorganisms in Sediment and Groundwater Are Implicated in Extracellular Redox Processes Based on Genomic Analysis of Bioanode Communities
Source: Front Microbiol. 2020 Jul 28;11:1694. doi: 10.3389/fmicb.2020.01694 (PMC7399161; doi:10.3389/fmicb.2020.01694)

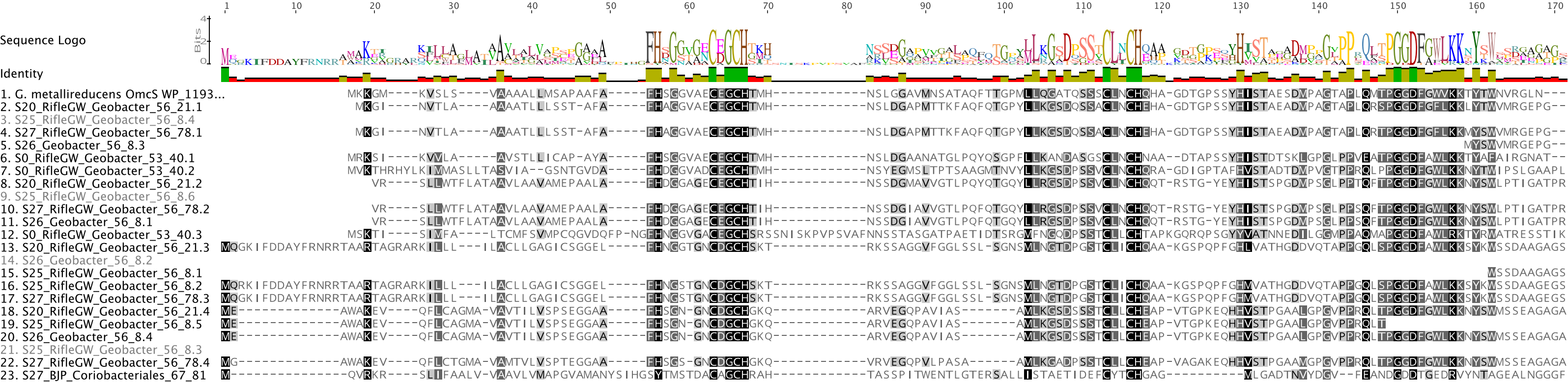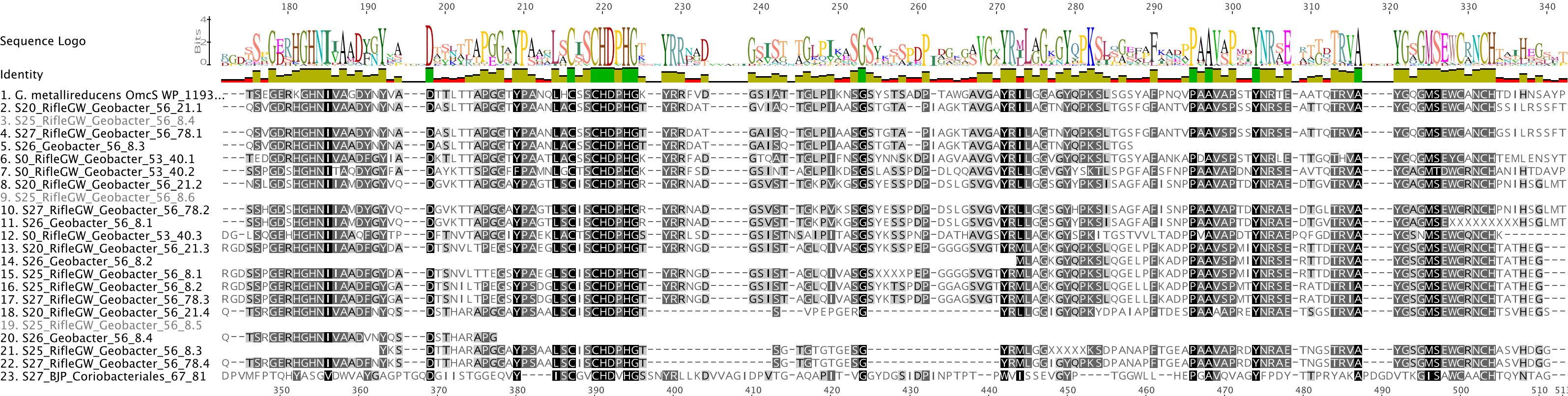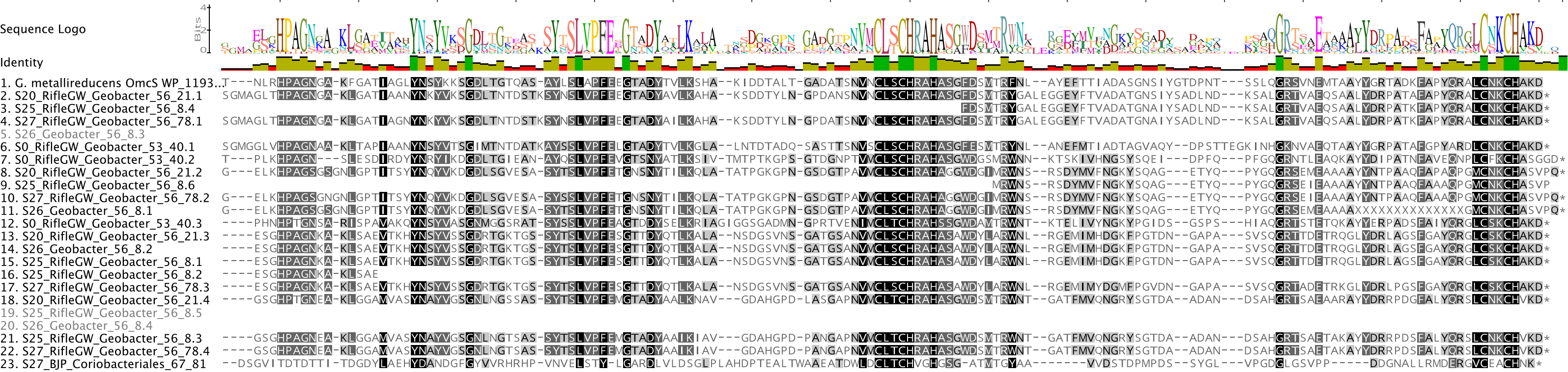

Supplement: Supplementary file 4 [file Data_Sheet_3.zip › FileS10_OmcS_homolog_align.pdf]

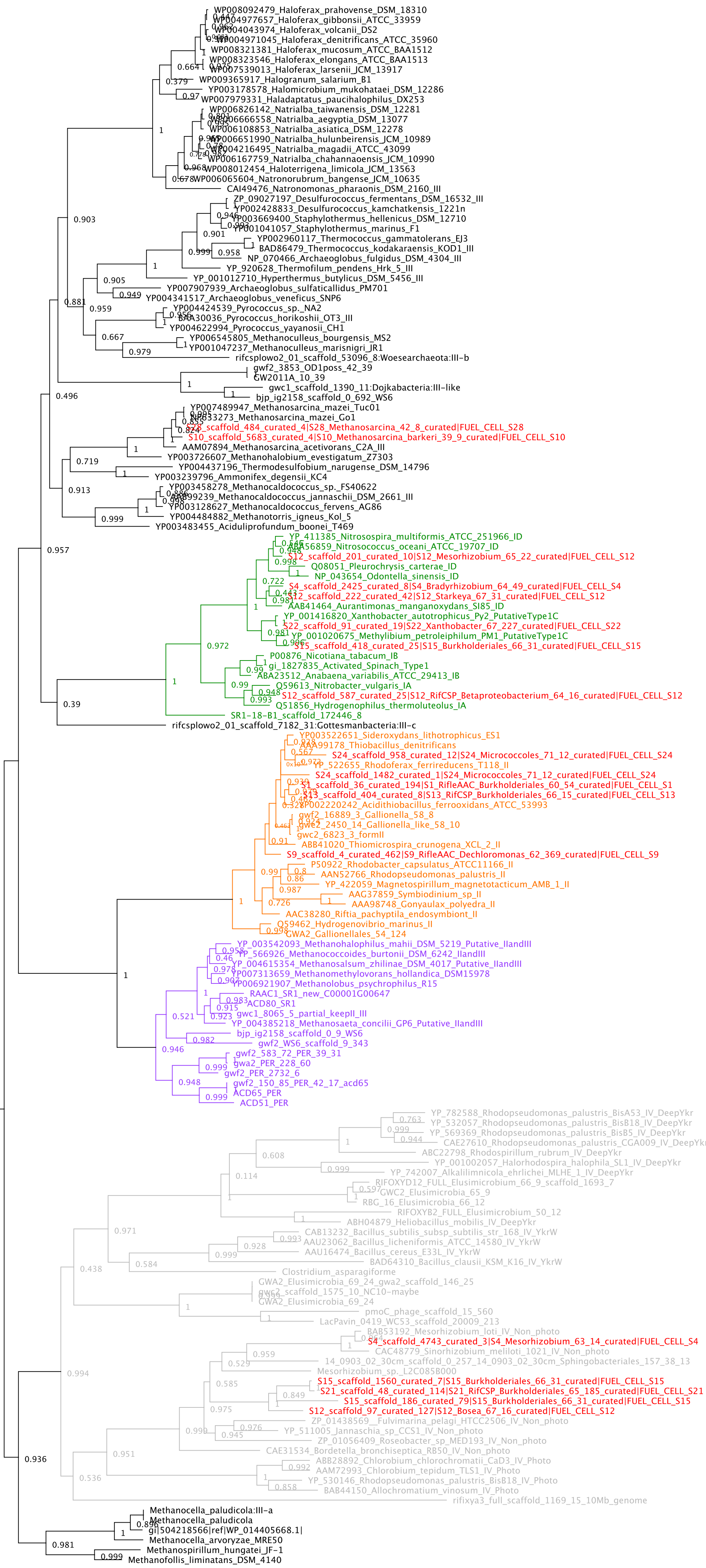

Supplement: Supplementary file 4 [file Data_Sheet_3.zip › FileS3_Rubisco_tree.pdf]
